# Supplementary material for: Engineering of CHO cells for the production of vertebrate recombinant sialyltransferases
Source: PeerJ. 2019 Feb 11;7:e5788. doi: 10.7717/peerj.5788 (PMC6375257; doi:10.7717/peerj.5788)
Supplement: Supplemental Information 1 — Edited sequences were aligned in MEGA-X and formatted for printing in GeneDoc. The L, S, III and VS motifs common to all SIATs are shown. Sequence differences from accession nos. are highlighted. Cys-184 (in full-length human ST6 Gal I) is shown with a blue arrow (paired Cys are shown in same colour). Sequence zST6 is foreshortened for alignment purposes–see Table 1 and Table S3 for full details. [file peerj-07-5788-s001.pdf]

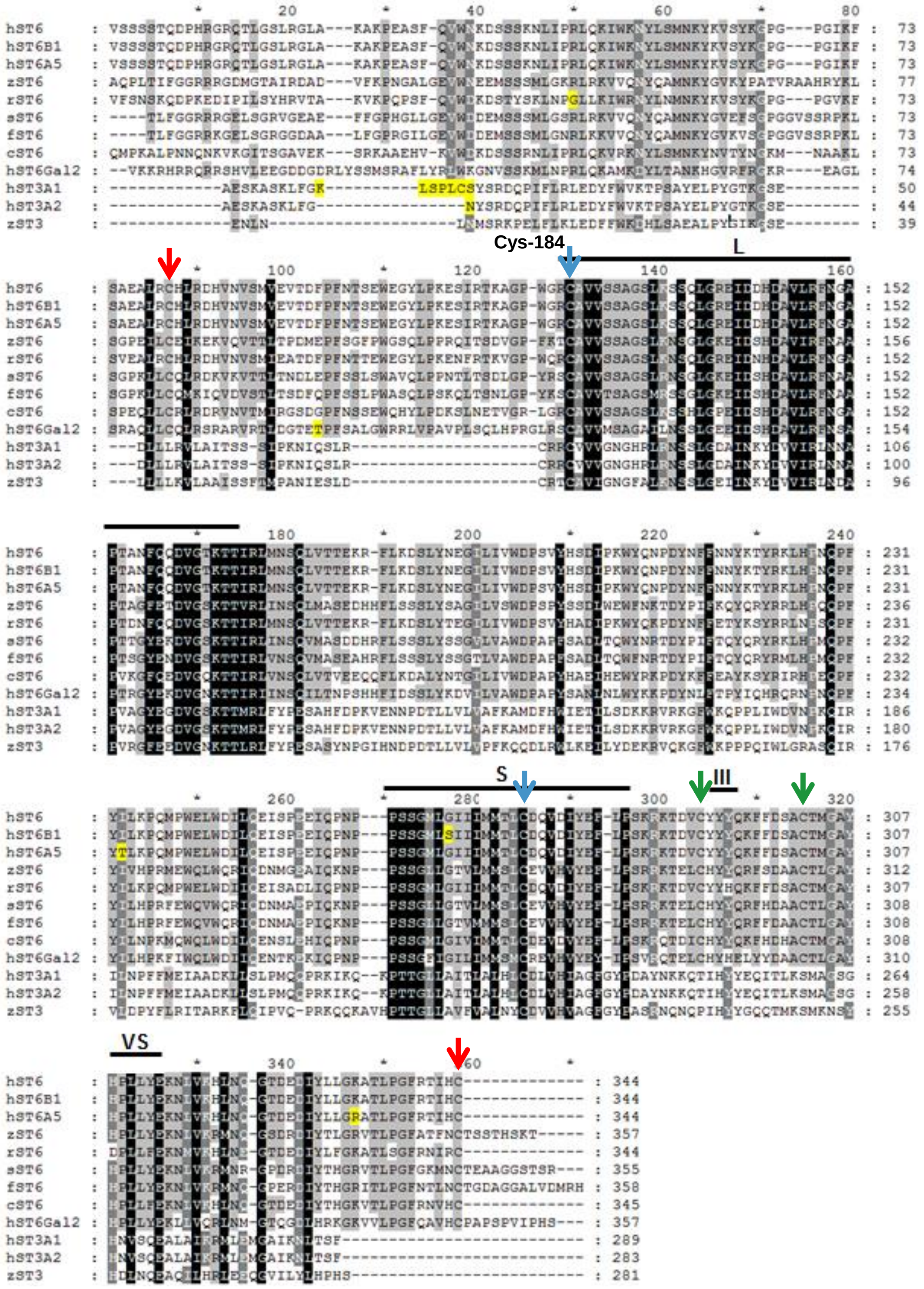


**Figure S1. Alignment of cloned SIAT catalytic domains**

Edited sequences were aligned in MEGA-X and formatted for printing in GeneDoc. The L, S, III and VS motifs common to all SIATs are shown. Sequence differences from accession nos. are highlighted. Cys-184 (in full-length human ST6 Gal I) is shown with a blue arrow (paired Cys are shown in same colour). Sequence zST6 is foreshortened for alignment purposes – see Table 1 and Table S3 for full details.
