# Supplementary material for: Engineering of CHO cells for the production of vertebrate recombinant sialyltransferases
Source: PeerJ. 2019 Feb 11;7:e5788. doi: 10.7717/peerj.5788 (PMC6375257; doi:10.7717/peerj.5788)
Supplement: Supplemental Information 2 — The choice of acceptor in any particular assay was determined by the assay format. For the routine sialyltransferase activity, the indirect phosphate linked assay, suitable acceptors were asialofetuin (ASF), LacNAc or Lac but as production of sialyllactose was the focus of our study, Lac or LacNAc were preferred over ASF. However, for lectin-binding assays, larger acceptor glycoprotein structures were more effective. Fetuin in its asialyated form, asialofetuin (ASF) is a commercially available glycoprotein which has N- and O-glycan chains offering eight possible terminal Gal sites. The schematic uses IUPAC glycan colour codes. [file peerj-07-5788-s002.pdf]

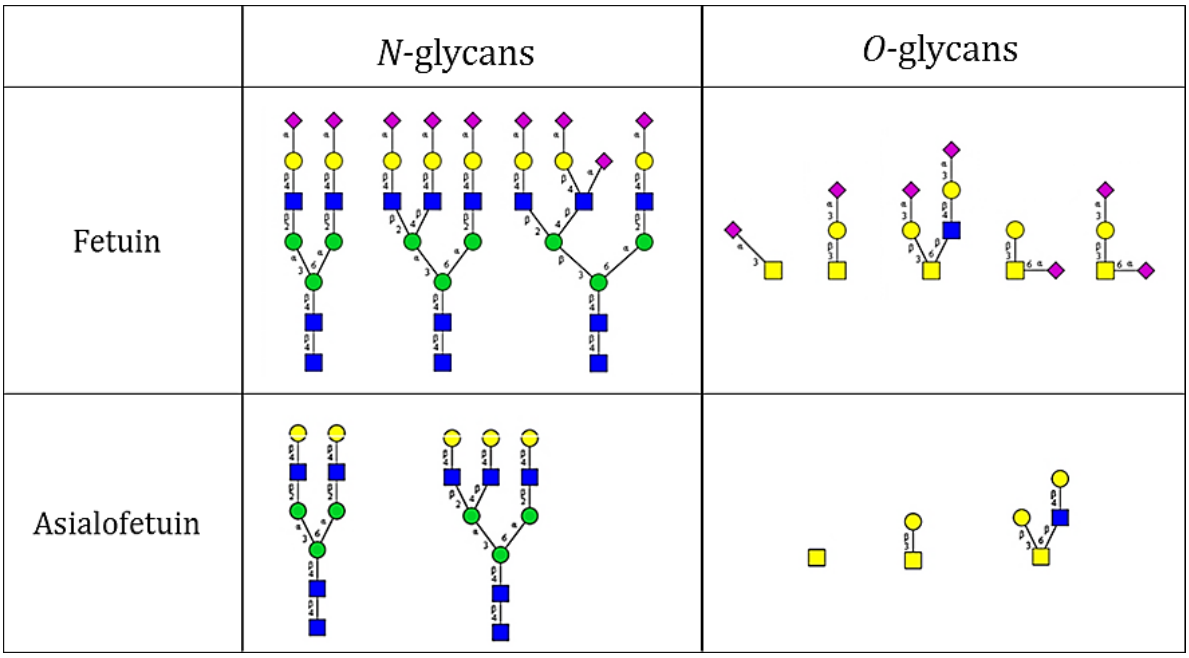

Figure S2: *O*- and *N*-glycosylation of fetuin and asialofetuin

The choice of acceptor in any particular assay was determined by the assay format. For the routine sialyltransferase activity, the indirect phosphate linked assay, suitable acceptors were asialofetuin (ASF), LacNAc or Lac but as production of sialyllactose was the focus of our study, Lac or LacNAc were preferred over ASF. However, for lectin-binding assays, larger acceptor glycoprotein structures were more effective. Fetuin in its asialyated form, asialofetuin (ASF) is a commercially available glycoprotein which has *N*- and *O*-glycan chains offering eight possible terminal Gal sites. The schematic uses IUPAC glycan colour codes.
