# Supplementary figures and images for: Engineering of CHO cells for the production of vertebrate recombinant sialyltransferases
Source: PeerJ. 2019 Feb 11;7:e5788. doi: 10.7717/peerj.5788 (PMC6375257; doi:10.7717/peerj.5788)

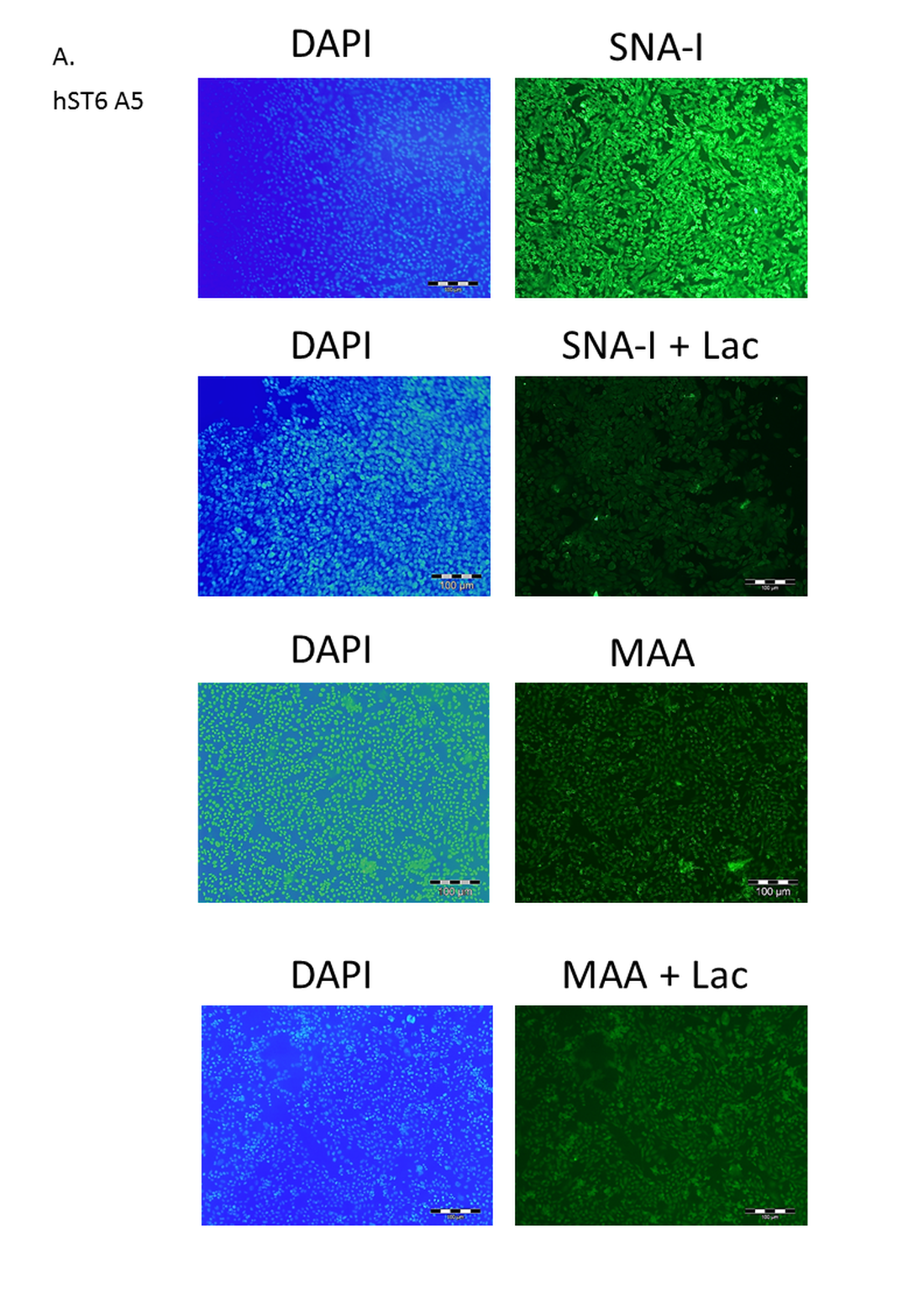

Supplement: Supplemental Information 3 — (A) CHO cells expressing ST6Gal I (hST6 A5) show strong binding of SNA-I and moderate binding of MAA. Lactose inhibits binding of SNA-I but has little effect on the binding of MAA. Lectins were FITC-labelled and cells were counter-stained with DAPI. Inhibition of binding of SNA-I was carried out in the presence of 100 mM lactose. [file peerj-07-5788-s003.png]

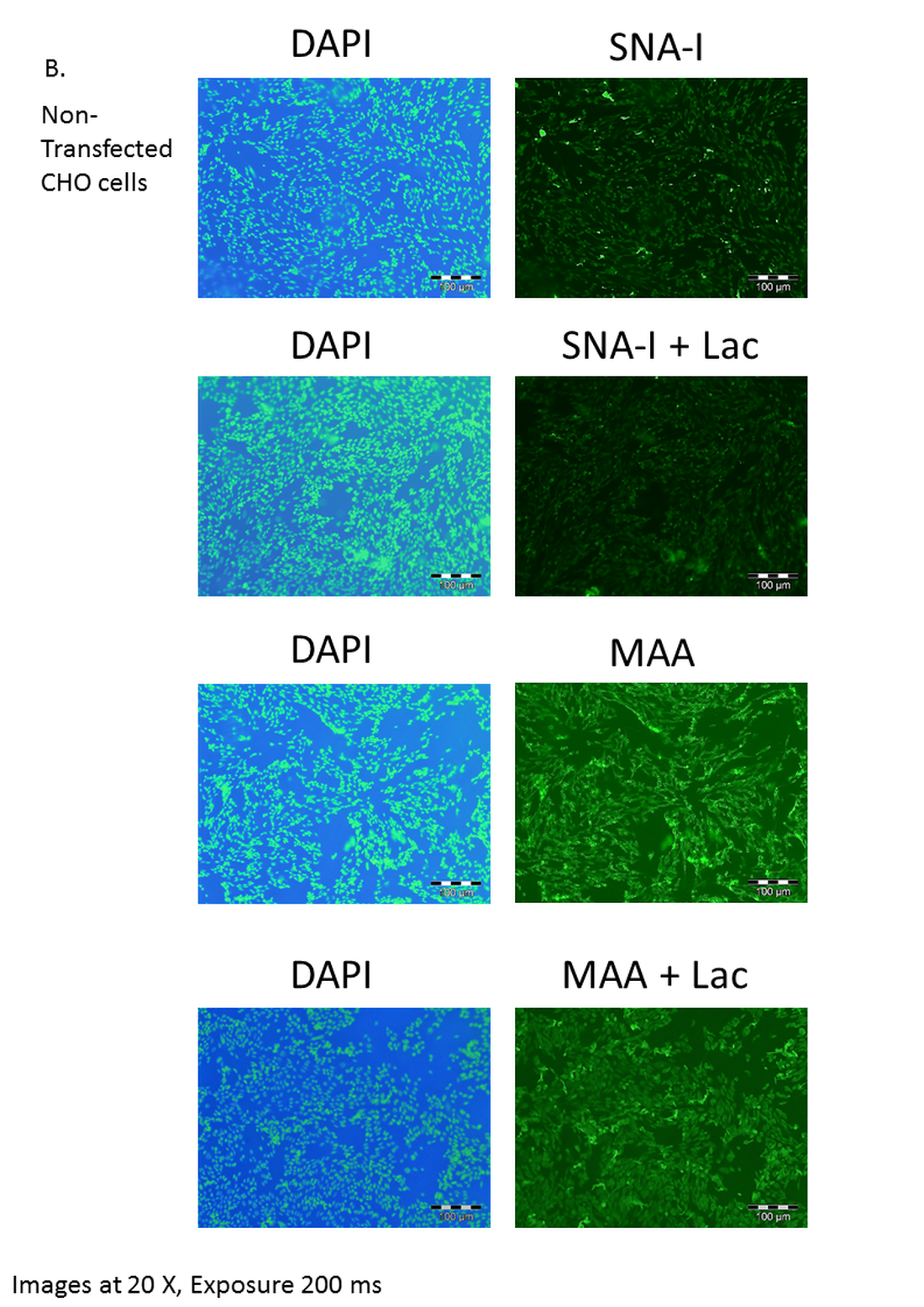

Supplement: Supplemental Information 4 — (B) CHO cells (non-transfected control) showed weak binding of SNA-I and moderate binding of MAA. Lactose has little effect on the binding of SNA-I or MAA. Lectins were FITC-labelled and cells were counter-stained with DAPI. Inhibition of binding of SNA-I was carried out in the presence of 100 mM lactose. [file peerj-07-5788-s004.png]

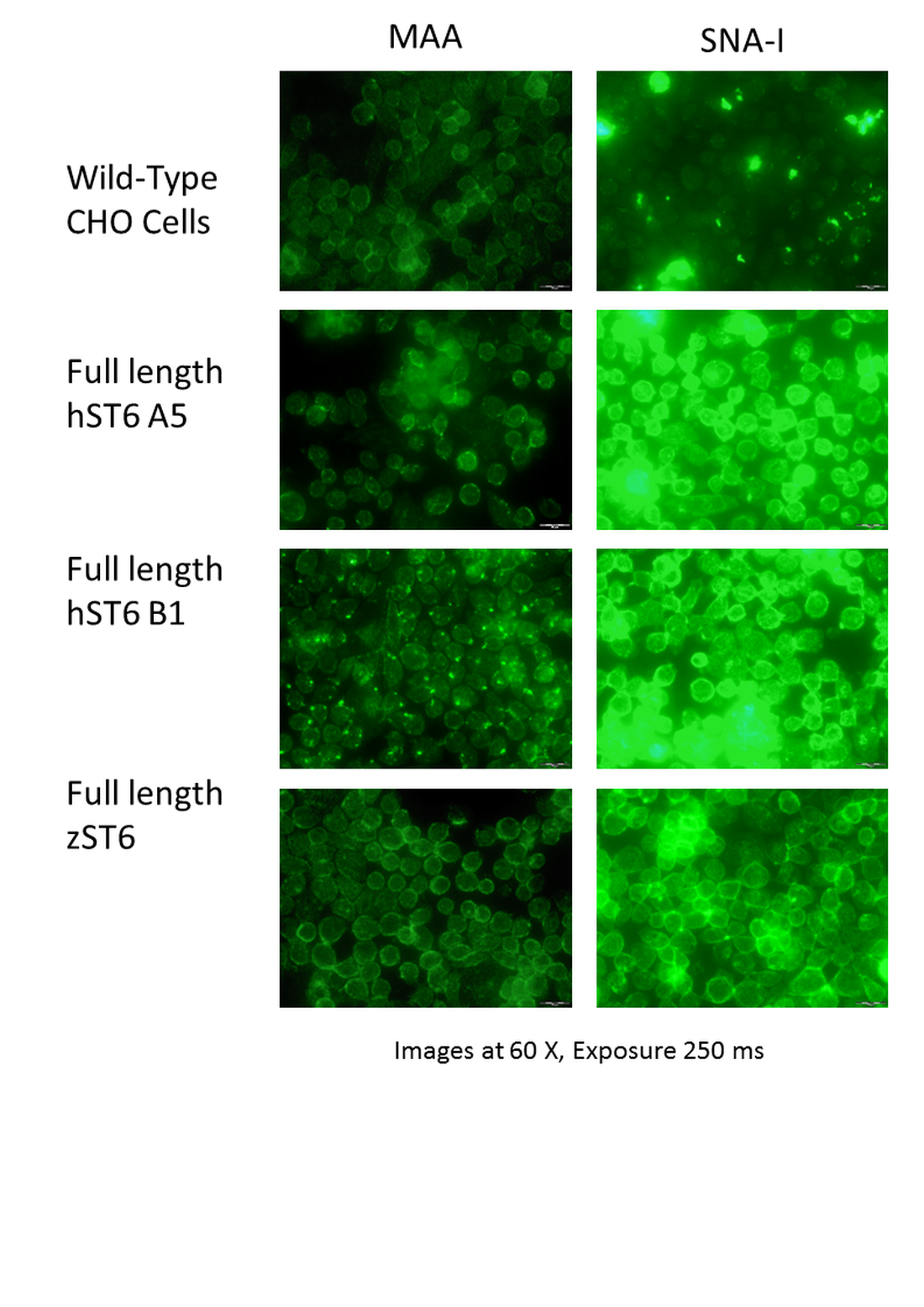

Supplement: Supplemental Information 5 — CHO cells (non-transfected controls) showed weak binding of SNA-I and moderate binding of MAA. Occasional cells showed strong binding to SNA-I. CHO cells expressing full-length ST6Gal I showed strong binding of SNA-I and moderate binding of MAA. Lectins were FITC-labelled. Constructs are named according to the equivalent catalytic constructs (see Table 1). [file peerj-07-5788-s005.png]
