# Supplementary material for: Engineering of CHO cells for the production of vertebrate recombinant sialyltransferases
Source: PeerJ. 2019 Feb 11;7:e5788. doi: 10.7717/peerj.5788 (PMC6375257; doi:10.7717/peerj.5788)
Supplement: Supplemental Information 6 — 1Construction of the zebrafish construct (zST6) was by direct TOPO cloning into pSecTag/FRT/V5-His-TOPO: there are therefore no corresponding attB1 or attB2 Gateway recombination sequences. Furthermore, although the catalytic domain starts at Val-63, similar to other ST6Gal proteins, much more of the stem is included in the final fusion (which is longer than most of the other cloned ST6Gal stems). [file peerj-07-5788-s006.docx]

**Table S1**. **Details of Flanking Sequences and Predicted Mr of Secreted Fusion Proteins** (split into N-terminal and C-terminal parts)

| **N-terminal Fusion** | | | | | | | |
| --- | --- | --- | --- | --- | --- | --- | --- |
| **Protein** | **Species** | **Clone ID** | **IgK secretion signal sequences in secreted protein** | **Mr** | **attB1/Gateway sequences** | **Mr** | **Mr SIAT** |
| ST3Gal4 | Human | hST3 A1 | AAQPARRARRTKLAL | 1679 | ITSLYKKAGSEFAL | 1528 | 32671 |
| ST3Gal4 | Human | hST3 A2 | AAQPARRARRTKLAL | 1679 | ITSLYKKAGSEFAL | 1528 | 32056 |
| ST3Gal4 | Zebrafish | zST3 | AAQPARRARRTKLAL | 1679 | ITSLYKKAGSEFALQKKW | 2098 | 32202 |
| ST6Gal1 | Human | hST6 | AAQPARRARRTKLAL | 1679 | ITSLYKKAGSEFAL | 1528 | 39498 |
| ST6Gal1 | Human | hST6 B1 | AAQPARRARRTKLAL | 1679 | ITSLYKKAGSEFAL | 1528 | 39528 |
| ST6Gal1 | Human | hST6 A5 | AAQPARRARRTKLAL | 1679 | ITSLYKKAGSEFAL | 1528 | 39514 |
| ST6Gal1 | Zebrafish^1^ | zST6 | AAQPARRARRTKLAL | 1679 |  | 0 | 47472 |
| ST6Gal1 | Rat | rST6 | AAQPARRARRTKLAL | 1679 | ITSLYKKAGSEFAL | 1528 | 39928 |
| ST6Gal1 | Stickleback | sST6 | AAQPARRARRTKLAL | 1679 | ITSLYKKAGS | 1067 | 39874 |
| ST6Gal1 | Fugu | fST6 | AAQPARRARRTKLAL | 1679 | ITSLYKKAGSEFAL | 1528 | 39960 |
| ST6Gal1 | Chicken | cST6 | AAQPARRARRTKLAL | 1679 | ITSLYKKAGSEFAL | 1528 | 39716 |
| ST6Gal2 | Human | hST6Gal2 | AAQPARRARRTKLAL | 1679 | ITSLYKKAGSEFAL | 1528 | 41019 |

^1^Construction of the zebrafish construct (zST6) was by direct TOPO cloning into pSecTag/FRT/V5-His-TOPO: there are therefore no corresponding attB1 or attB2 Gateway recombination sequences. Furthermore, although the catalytic domain starts at Val-63, similar to other ST6Gal proteins, much more of the stem is included in the final fusion (which is longer than most of the other cloned ST6Gal stems).
