# Supplementary material for: Engineering of CHO cells for the production of vertebrate recombinant sialyltransferases
Source: PeerJ. 2019 Feb 11;7:e5788. doi: 10.7717/peerj.5788 (PMC6375257; doi:10.7717/peerj.5788)
Supplement: Supplemental Information 7 — 1The clone name (ST6 for ST6Gal1, ST3 for ST3Gal4) is prefixed by the species (h, human; z, zebrafish; r, rat; s, stickleback; f, fugu; c, chicken) and suffixes refer to different forms. [file peerj-07-5788-s007.docx]

**Table S2. Sialyltransferases source detail**

| **Protein** | **Clone name^1^** | **Species** | **Habitat/Temperature** | **RNA source** |
| --- | --- | --- | --- | --- |
| ST3Gal4 | hST3 A1 | *Homo sapiens* | Homoeothermic - 37^o^C | HepG2 cell line |
| ST3Gal4 | hST3 A2 | *Homo sapiens* | Homoeothermic - 37^o^C | HepG2 cell line |
| ST3Gal4 | zST3 | *Danio rerio* | Poikilothermic – 18 - 28^o^C (limits: 6 - 41^o^C) | Head |
| ST6Gal1 | hST6 | *Homo sapiens* | Homoeothermic - 37^o^C | HepG2 cell line |
| ST6Gal1 | hST6 B1 | *Homo sapiens* | Homoeothermic - 37^o^C | HepG2 cell line |
| ST6Gal1 | hST6 A5 | *Homo sapiens* | Homoeothermic - 37^o^C | HepG2 cell line |
| ST6Gal1 | zST6 | *Danio rerio* | Poikilothermic – 18 - 28^o^C (limits: 6 - 41^o^C) | Head |
| ST6Gal1 | rST6 | *Rattus norvegicus* | Homoeothermic - 37^o^C | Brain |
| ST6Gal1 | sST6 | *Gasterosteus aculeatus* | Poikilothermic – Sea/Freshwater 4 - 22^o^C (limits: 0 - 29^o^C) | Head |
| ST6Gal1 | fST6 | *Takifugu rubripes* | Poikilothermic- Seawater 15 - 24^o^C (limits: 0 - 30^o^C) | Head |
| ST6Gal1 | cST6 | *Gallus gallus* | Homoeothermic - 42^o^C | Spleen |
| ST6Gal2 | hST6Gal2 | *Homo sapiens* | Homoeothermic - 37^o^C | RPMI cell line |

^1^ The clone name (ST6 for ST6Gal1, ST3 for ST3Gal4) is prefixed by the species (h, human; z, zebrafish; r, rat; s, stickleback; f, fugu; c, chicken) and suffixes refer to different forms.
