# Supplementary material for: Engineering of CHO cells for the production of vertebrate recombinant sialyltransferases
Source: PeerJ. 2019 Feb 11;7:e5788. doi: 10.7717/peerj.5788 (PMC6375257; doi:10.7717/peerj.5788)
Supplement: Supplemental Information 8 — 1Where the sequence differs from the accession the amino acid is marked in red text and highlighted. The corresponding native sequence (if included) is highlighted. [file peerj-07-5788-s008.docx]

**Table S3. Source sequences and detail on secreted protein**

| **Clone name** | **Accession** | **Mr (Da)**  **Secreted fusion** | **Catalytic start** | **AA of SIAT fusion** | **Sequence^1^** |
| --- | --- | --- | --- | --- | --- |
| hST3 A1 | AF525084_1 | 41166 | Ala-47 | 289 | AESKASKLFGKLSPLCSYSRDQPIFLRLEDYFWVKTPSAYELPYGTKGSEDLLLRVLAITSSSIPKNIQSLRCRRCVVVGNGHRLRNSSLGDAINKYDVVIRLNNAPVAGYEGDVGSKTTMRLFYPESAHFDPKVENNPDTLLVLVAFKAMDFHWIETILSDKKRVRKGFWKQPPLIWDVNPKQIRILNPFFMEIAADKLLSLPMQQPRKIKQKPTTGLLAITLALHLCDLVHIAGFGYPDAYNKKQTIHYYEQITLKSMAGSGHNVSQEALAIKRMLEMGAIKNLTSF |
| hST3 A2 | NP_001241686 | 40551 | Ala-47 | 283 | AESKASKLFGNYSRDQPIFLRLEDYFWVKTPSAYELPYGTKGSEDLLLRVLAITSSSIPKNIQSLRCRRCVVVGNGHRLRNSSLGDAINKYDVVIRLNNAPVAGYEGDVGSKTTMRLFYPESAHFDPKVENNPDTLLVLVAFKAMDFHWIETILSDKKRVRKGFWKQPPLIWDVNPKQIRILNPFFMEIAADKLLSLPMQQPRKIKQKPTTGLLAITLALHLCDLVHIAGFGYPDAYNKKQTIHYYEQITLKSMAGSGHNVSQEALAIKRMLEMGAIKNLTSF |
| zST3 | NP_001076498 | 39169 | Glu-55 | 281 | ENLNLNMSRKPELFLKLEDFFWKDHLSAEALPYGIKGSELLLLKVLAAISSFTMPANIESLDCRTCAVIGNGFALKNSSLGEIINKYDVVIRLNDAPVRGFEEDVGNKTTLRLFYPESASYNPGIHNDPDTLLVLVPFKQQDLRWLKEILYDEKRVQKGFWKPPPQIWLGRASQIRVLDPYFLRITARKFLQIPVQPRKQQKAVHPTTGLLAVFVALNYCDVVHVAGFGYPASRNQNQPIHYYGQQTMKSMKNSYHDLNQEAQILHRLEEQGVILYLHPHS |
| hST6 | NP_003023.1 | 47993 | Val-63 | 344 | VSSSSTQDPHRGRQTLGSLRGLAKAKPEASFQVWNKDSSSKNLIPRLQKIWKNYLSMNKYKVSYKGPGPGIKFSAEALRCHLRDHVNVSMVEVTDFPFNTSEWEGYLPKESIRTKAGPWGRCAVVSSAGSLKSSQLGREIDDHDAVLRFNGAPTANFQQDVGTKTTIRLMNSQLVTTEKRFLKDSLYNEGILIVWDPSVYHSDIPKWYQNPDYNFFNNYKTYRKLHPNQPFYILKPQMPWELWDILQEISPEEIQPNPPSSGMLGIIIMMTLCDQVDIYEFLPSKRKTDVCYYYQKFFDSACTMGAYHPLLYEKNLVKHLNQGTDEDIYLLGKATLPGFRTIHC |
| hST6 B1 | NP_003023.1 | 48023 | Val-63 | 344 | VSSSSTQDPHRGRQTLGSLRGLAKAKPEASFQVWNKDSSSKNLIPRLQKIWKNYLSMNKYKVSYKGPGPGIKFSAEALRCHLRDHVNVSMVEVTDFPFNTSEWEGYLPKESIRTKAGPWGRCAVVSSAGSLKSSQLGREIDDHDAVLRFNGAPTANFQQDVGTKTTIRLMNSQLVTTEKRFLKDSLYNEGILIVWDPSVYHSDIPKWYQNPDYNFFNNYKTYRKLHPNQPFYILKPQMPWELWDILQEISPEEIQPNPPSSGMLSIIIMMTLCDQVDIYEFLPSKRKTDVCYYYQKFFDSACTMGAYHPLLYEKNLVKHLNQGTDEDIYLLGKATLPGFRTIHC |
| hST6 A5 | NP_003023.1 | 48009 | Val-63 | 344 | VSSSSTQDPHRGRQTLGSLRGLAKAKPEASFQVWNKDSSSKNLIPRLQKIWKNYLSMNKYKVSYKGPGPGIKFSAEALRCHLRDHVNVSMVEVTDFPFNTSEWEGYLPKESIRTKAGPWGRCAVVSSAGSLKSSQLGREIDDHDAVLRFNGAPTANFQQDVGTKTTIRLMNSQLVTTEKRFLKDSLYNEGILIVWDPSVYHSDIPKWYQNPDYNFFNNYKTYRKLHPNQPFYTLKPQMPWELWDILQEISPEEIQPNPPSSGMLGIIIMMTLCDQVDIYEFLPSKRKTDVCYYYQKFFDSACTMGAYHPLLYEKNLVKHLNQGTDEDIYLLGRATLPGFRTIHC |
| zST6 | NP_001003853.1 | 52811 | Val-63 | 422 | VKVLRGTGGSKPMYTDPQKLPGVIPGDPQKPIPILSSSNYSMESTSKDISLGGKKRERGLFYWLLAQPLTIFGGRRRGDMGTAIRDADVFKPNGALGEVWNEEMSSSMLGKRLRKVVQNYQAMNKYGVKYPATVRAAHRYKLSGPEILCEIKEKVQVTTLTPDMEPFSGFPWGSQLPPRQITSDVGPFKTCAVVSSAGSLKNSGLGKEIDSHDAVIRFNAAPTAGFETDVGSKTTVRLINSQLMASEDHHFLSSSLYSAGILVSWDPSPYSSDLWEWFNKTDYPIFKQYQRYRRLHPQQPFYIVHPRMEWQLWQRIQDNMGEAIQKNPPSSGLLGTVLMMSLCEVVHVYEFLPSRRKTELCHYYQRFSDAACTLGAYHPLLYEKNLVKRMNQGSDRDIYTLGRVTLPGFATFNCTSSTHSKT |
| rST6 | P13721.1 | 48422 | Val-60 | 344 | VFSNSKQDPKEDIPILSYHRVTAKVKPQPSFQVWDKDSTYSKLNPGLLKIWRNYLNMNKYKVSYKGPGPGVKFSVEALRCHLRDHVNVSMIEATDFPFNTTEWEGYLPKENFRTKVGPWQRCAVVSSAGSLKNSQLGREIDNHDAVLRFNGAPTDNFQQDVGSKTTIRLMNSQLVTTEKRFLKDSLYTEGILIVWDPSVYHADIPKWYQKPDYNFFETYKSYRRLNPSQPFYILKPQMPWELWDIIQEISADLIQPNPPSSGMLGIIIMMTLCDQVDIYEFLPSKRKTDVCYYHQKFFDSACTMGAYDPLLFEKNMVKHLNEGTDEDIYLFGKATLSGFRNIRC |
| sST6 | CBQ74103.1 | 47495 | Thr-136 | 355 | TLFGGRRRGELSGRVGEAEFFGPHGLLGEVWDDEMSSSMLGSRLRKVVQNYQAMNKYGVEFSGPGGVSSRPKLSGPKLLCQLRDKVKVTTLTNDLEPFSSLSWAVQLPPNTLTSDLGPYRSCAVVSSAGSLRNSGLGKEIDSHDAVLRFNAAPTTGYEKDVGSKTTIRLINSQVMASDDHRFLSSSLYSSGVLVAWDPAPFSADLTQWYNRTDYPIFTQYQRYRKLHPMQPFYILHPRFEWQVWQRIQDNMAEPIQKNPPSSGLLGTVLMMSLCEVVHVYEFLPSRRKTELCHYYQRFHDAACTLGAYHPLLYEKNLVKRMNRGPDRDIYTHGRVTLPGFGKMNCTEAAGGSTSR |
| fST6 | NP_001027933.1 | 48454 | Thr-136 | 358 | TLFGGRRKGELSGRGGDAALFGPRGILGEVWDDEMSSSMLGNRLKKVVQNYQAMNKYGVKVSGPGGVSSRPKLSGPKLLCQMKIQVDVSTLTSDFQPFSSLPWASQLPSKQLTSNLGPYKSCAVVTSAGSMRSSGLGKEIDSHDAVLRFNAAPTSGYENDVGSKTTIRLVNSQVMASEAHRFLSSSLYSSGTLVAWDPAPFSADLTQWFNRTDYPIFTQYQRYRMLHPMQPFYILHPRFEWQVWQRIQDNMAEPIQKNPPSSGLLGTVMMMSLCEVVHVYEFLPSRRKTELCHYYQRFFDAACTLGAYHPLLYEKNLVKRMNQGPERDIYTHGRITLPGFNTLNCTGDAGGALVDMRH |
| cST6 | XP_015132322 | 48211 | Gln-69 | 345 | QMPKALPNNQNKVKGITSGAVEKSRKAAEHVKVWDKDSSSRNLIPRLQKVRKNYLSMNKYNVTYNGKMNAAKLSPEQLLCRLRDRVNVTMIRGSDGPFNSSEWQHYLPDKSLNETVGRLGRCAVVSSAGSLKSSHLGPEIDSHDAVLRFNGAPVKGFQEDVGQKTTIRLVNSQLVTVEEQQFLKDALYNTGILIVWDPAPYHAEIHEWYRKPDYKFFEAYKSYRIRHPEQPFYILNPKMQWQLWDILQENSLEHIQPNPPSSGMLGIVIMMTLCDEVDVYEFLPSKRQTDICHYYQKFHDHACTMGAYHPLLFEKNLVKHLNQGTDEDIYTHGKVTLPGFRNVHC |
| hST6Gal2 | NP_115917 | 49514 | Val-173 | 357 | VKKRHRRQRRSHVLEEGDDGDRLYSSMSRAFLYRLWKGNVSSKMLNPRLQKAMKDYLTANKHGVRFRGKREAGLSRAQLLCQLRSRARVRTLDGTETPFSALGWRRLVPAVPLSQLHPRGLRSCAVVMSAGAILNSSLGEEIDSHDAVLRFNSAPTRGYEKDVGNKTTIRIINSQILTNPSHHFIDSSLYKDVILVAWDPAPYSANLNLWYKKPDYNLFTPYIQHRQRNPNQPFYILHPKFIWQLWDIIQENTKEKIQPNPPSSGFIGILIMMSMCREVHVYEYIPSVRQTELCHYHELYYDAACTLGAYHPLLYEKLLVQRLNMGTQGDLHRKGKVVLPGFQAVHCPAPSPVIPHS |

^1^ Where the sequence differs from the accession the amino acid is marked in red text and highlighted. The corresponding native sequence (if included) is highlighted.
