# Supplementary material for: Engineering of CHO cells for the production of vertebrate recombinant sialyltransferases
Source: PeerJ. 2019 Feb 11;7:e5788. doi: 10.7717/peerj.5788 (PMC6375257; doi:10.7717/peerj.5788)

Figure 3: Statistics

Univariate Analysis of Variance

Legend to Figure 3 Raw Data Set 2

Label for Statistics	Corresponding in Manuscript
A5	hST6 A5
B1	hST6 B1
G1	hST6
Dre	zST6
Gac	sST6
Tru	fST6
Gga	cST6
Rno	rST6
Ctrl	mST6 (commercial mouse enzyme)
ST3A2	hST3 A2
ST3A1	hST3 A2
ST3H6	zST3
ST2	hST6Gal2
Neg	Neg (IMAC-purified PSA[1])

[1]PSA: Prostate Specific Antigen (no sialyltransferase activity)

Notes

Output Created		12-JUL-2018 10:38:56
Comments		
Input	Data	\\gweng.gmadtree.gmit.ie\STAFF\Benoit.Houeix\Documents\ST.sav
	Active Dataset	DataSet1
	Filter	<none>
	Weight	<none>
	Split File	<none>
	N of Rows in Working Data File	48
Missing Value Handling	Definition of Missing	User-defined missing values are treated as missing.

	Cases Used	Statistics are based on all cases with valid data for all variables in the model.
Syntax		UNIANOVA Activity BY Sample<br>/METHOD=SSTYPE(3)<br>/INTERCEPT=INCLUDE<br>/POSTHOC=Sample(BONFERRONI)<br>/PLOT=PROFILE(Sample)<br>/EMMEANS=TABLES(Sample)<br>/PRINT=HOMOGENEITY DESCRIPTIVE<br>/CRITERIA=ALPHA(.05)<br>/DESIGN=Sample.
Resources	Processor Time	00:00:00.92
	Elapsed Time	00:00:00.62

Between-Subjects Factors

		Value Label	N
Sample	A5	A5	3
	B1	B1	3
	Ctrl	Ctrl	3
	Dre	Dre	3
	G1	G1	3
	Gac	Gac	3
	Gga	Gga	3
	Neg	Neg	3
	Rno	Rno	3
	ST2	ST2	3
	ST3A1	ST3A1	3
	ST3A2	ST3A2	3
	ST3H6	ST3H6	3
	Tru	Tru	3

Descriptive Statistics

Note: These means are pmol/well, NOT pmol/min/µg protein as presented in Figure 3.

Dependent Variable: Activity

Sample	Mean	Std. Deviation	N
A5	2197.3667	312.12468	3

B1	2440.0533	584.34650	3
Ctrl	2451.6167	455.31970	3
Dre	2660.1367	397.39347	3
G1	2620.6967	345.92251	3
Gac	3065.8800	432.52984	3
Gga	2011.2967	280.50808	3
Neg	199.0700	243.39219	3
Rno	3126.6500	659.51091	3
ST2	853.5867	108.60899	3
ST3A1	885.0133	115.04271	3
ST3A2	793.7633	102.81655	3
ST3H6	2592.9033	572.48364	3
Tru	3247.6667	434.25077	3
Total	2081.8357	1020.18187	42

Levene's Test of Equality of Error Variances[a]

Dependent Variable: Activity

F	df1	df2	Sig.
1.532	13	28	.167

Tests the null hypothesis that the error variance of the dependent variable is equal across groups.[a]

a. Design: Intercept + Sample

Tests of Between-Subjects Effects

Dependent Variable: Activity

Source	Type III Sum of Squares	df	Mean Square	F	Sig.
Corrected Model	38200298.363[a]	13	2938484.489	18.401	.000
Intercept	182029677.534	1	182029677.534	1139.896	.000
Sample	38200298.363	13	2938484.489	18.401	.000
Error	4471314.225	28	159689.794		
Total	224701290.122	42			
Corrected Total	42671612.588	41			

a. R Squared = .895 (Adjusted R Squared = .847)

Estimated Marginal Means

Sample

Dependent Variable: Activity

Sample	Mean	Std. Error	95% Confidence Interval

			Lower Bound	Upper Bound
A5	2197.367	230.716	1724.766	2669.967
B1	2440.053	230.716	1967.453	2912.654
Ctrl	2451.617	230.716	1979.016	2924.217
Dre	2660.137	230.716	2187.536	3132.737
G1	2620.697	230.716	2148.096	3093.297
Gac	3065.880	230.716	2593.279	3538.481
Gga	2011.297	230.716	1538.696	2483.897
Neg	199.070	230.716	-273.531	671.671
Rno	3126.650	230.716	2654.049	3599.251
ST2	853.587	230.716	380.986	1326.187
ST3A1	885.013	230.716	412.413	1357.614
ST3A2	793.763	230.716	321.163	1266.364
ST3H6	2592.903	230.716	2120.303	3065.504
Tru	3247.667	230.716	2775.066	3720.267

Post Hoc Tests

Sample

Multiple Comparisons

Dependent Variable: Activity

Bonferroni

(I) Sample	(J) Sample	Mean Difference (I-J)	Std. Error	Sig.	95% Confidence Interval	
					Lower Bound	Upper Bound
A5	B1	-242.6867	326.28188	1.000	-1515.1029	1029.7296
	Ctrl	-254.2500	326.28188	1.000	-1526.6662	1018.1662
	Dre	-462.7700	326.28188	1.000	-1735.1862	809.6462
	G1	-423.3300	326.28188	1.000	-1695.7462	849.0862
	Gac	-868.5133	326.28188	1.000	-2140.9296	403.9029
	Gga	186.0700	326.28188	1.000	-1086.3462	1458.4862
	Neg	1998.2967*	326.28188	.000	725.8804	3270.7129
	Rno	-929.2833	326.28188	.742	-2201.6996	343.1329
	ST2	1343.7800*	326.28188	.028	71.3638	2616.1962
	ST3A1	1312.3533*	326.28188	.036	39.9371	2584.7696
	ST3A2	1403.6033*	326.28188	.017	131.1871	2676.0196
	ST3H6	-395.5367	326.28188	1.000	-1667.9529	876.8796
	Tru	-1050.3000	326.28188	.295	-2322.7162	222.1162
B1	A5	242.6867	326.28188	1.000	-1029.7296	1515.1029
	Ctrl	-11.5633	326.28188	1.000	-1283.9796	1260.8529
	Dre	-220.0833	326.28188	1.000	-1492.4996	1052.3329

	G1	-180.6433	326.28188	1.000	-1453.0596	1091.7729
	Gac	-625.8267	326.28188	1.000	-1898.2429	646.5896
	Gga	428.7567	326.28188	1.000	-843.6596	1701.1729
	Neg	2240.9833*	326.28188	.000	968.5671	3513.3996
	Rno	-686.5967	326.28188	1.000	-1959.0129	585.8196
	ST2	1586.4667*	326.28188	.004	314.0504	2858.8829
	ST3A1	1555.0400*	326.28188	.005	282.6238	2827.4562
	ST3A2	1646.2900*	326.28188	.002	373.8738	2918.7062
	ST3H6	-152.8500	326.28188	1.000	-1425.2662	1119.5662
	Tru	-807.6133	326.28188	1.000	-2080.0296	464.8029
Ctrl	A5	254.2500	326.28188	1.000	-1018.1662	1526.6662
	B1	11.5633	326.28188	1.000	-1260.8529	1283.9796
	Dre	-208.5200	326.28188	1.000	-1480.9362	1063.8962
	G1	-169.0800	326.28188	1.000	-1441.4962	1103.3362
	Gac	-614.2633	326.28188	1.000	-1886.6796	658.1529
	Gga	440.3200	326.28188	1.000	-832.0962	1712.7362
	Neg	2252.5467*	326.28188	.000	980.1304	3524.9629
	Rno	-675.0333	326.28188	1.000	-1947.4496	597.3829
	ST2	1598.0300*	326.28188	.003	325.6138	2870.4462
	ST3A1	1566.6033*	326.28188	.004	294.1871	2839.0196
	ST3A2	1657.8533*	326.28188	.002	385.4371	2930.2696
	ST3H6	-141.2867	326.28188	1.000	-1413.7029	1131.1296
	Tru	-796.0500	326.28188	1.000	-2068.4662	476.3662
Dre	A5	462.7700	326.28188	1.000	-809.6462	1735.1862
	B1	220.0833	326.28188	1.000	-1052.3329	1492.4996
	Ctrl	208.5200	326.28188	1.000	-1063.8962	1480.9362
	G1	39.4400	326.28188	1.000	-1232.9762	1311.8562
	Gac	-405.7433	326.28188	1.000	-1678.1596	866.6729
	Gga	648.8400	326.28188	1.000	-623.5762	1921.2562
	Neg	2461.0667*	326.28188	.000	1188.6504	3733.4829
	Rno	-466.5133	326.28188	1.000	-1738.9296	805.9029
	ST2	1806.5500*	326.28188	.001	534.1338	3078.9662
	ST3A1	1775.1233*	326.28188	.001	502.7071	3047.5396
	ST3A2	1866.3733*	326.28188	.000	593.9571	3138.7896
	ST3H6	67.2333	326.28188	1.000	-1205.1829	1339.6496
	Tru	-587.5300	326.28188	1.000	-1859.9462	684.8862
G1	A5	423.3300	326.28188	1.000	-849.0862	1695.7462
	B1	180.6433	326.28188	1.000	-1091.7729	1453.0596
	Ctrl	169.0800	326.28188	1.000	-1103.3362	1441.4962
	Dre	-39.4400	326.28188	1.000	-1311.8562	1232.9762
	Gac	-445.1833	326.28188	1.000	-1717.5996	827.2329

	Gga	609.4000	326.28188	1.000	-663.0162	1881.8162
	Neg	2421.6267*	326.28188	.000	1149.2104	3694.0429
	Rno	-505.9533	326.28188	1.000	-1778.3696	766.4629
	ST2	1767.1100*	326.28188	.001	494.6938	3039.5262
	ST3A1	1735.6833*	326.28188	.001	463.2671	3008.0996
	ST3A2	1826.9333*	326.28188	.000	554.5171	3099.3496
	ST3H6	27.7933	326.28188	1.000	-1244.6229	1300.2096
	Tru	-626.9700	326.28188	1.000	-1899.3862	645.4462
Gac	A5	868.5133	326.28188	1.000	-403.9029	2140.9296
	B1	625.8267	326.28188	1.000	-646.5896	1898.2429
	Ctrl	614.2633	326.28188	1.000	-658.1529	1886.6796
	Dre	405.7433	326.28188	1.000	-866.6729	1678.1596
	G1	445.1833	326.28188	1.000	-827.2329	1717.5996
	Gga	1054.5833	326.28188	.286	-217.8329	2326.9996
	Neg	2866.8100*	326.28188	.000	1594.3938	4139.2262
	Rno	-60.7700	326.28188	1.000	-1333.1862	1211.6462
	ST2	2212.2933*	326.28188	.000	939.8771	3484.7096
	ST3A1	2180.8667*	326.28188	.000	908.4504	3453.2829
	ST3A2	2272.1167*	326.28188	.000	999.7004	3544.5329
	ST3H6	472.9767	326.28188	1.000	-799.4396	1745.3929
	Tru	-181.7867	326.28188	1.000	-1454.2029	1090.6296
Gga	A5	-186.0700	326.28188	1.000	-1458.4862	1086.3462
	B1	-428.7567	326.28188	1.000	-1701.1729	843.6596
	Ctrl	-440.3200	326.28188	1.000	-1712.7362	832.0962
	Dre	-648.8400	326.28188	1.000	-1921.2562	623.5762
	G1	-609.4000	326.28188	1.000	-1881.8162	663.0162
	Gac	-1054.5833	326.28188	.286	-2326.9996	217.8329
	Neg	1812.2267*	326.28188	.001	539.8104	3084.6429
	Rno	-1115.3533	326.28188	.177	-2387.7696	157.0629
	ST2	1157.7100	326.28188	.127	-114.7062	2430.1262
	ST3A1	1126.2833	326.28188	.163	-146.1329	2398.6996
	ST3A2	1217.5333	326.28188	.078	-54.8829	2489.9496
	ST3H6	-581.6067	326.28188	1.000	-1854.0229	690.8096
	Tru	-1236.3700	326.28188	.067	-2508.7862	36.0462
Neg	A5	-1998.2967*	326.28188	.000	-3270.7129	-725.8804
	B1	-2240.9833*	326.28188	.000	-3513.3996	-968.5671
	Ctrl	-2252.5467*	326.28188	.000	-3524.9629	-980.1304
	Dre	-2461.0667*	326.28188	.000	-3733.4829	-1188.6504
	G1	-2421.6267*	326.28188	.000	-3694.0429	-1149.2104
	Gac	-2866.8100*	326.28188	.000	-4139.2262	-1594.3938
	Gga	-1812.2267*	326.28188	.001	-3084.6429	-539.8104

	Rno	-2927.5800*	326.28188	.000	-4199.9962	-1655.1638
	ST2	-654.5167	326.28188	1.000	-1926.9329	617.8996
	ST3A1	-685.9433	326.28188	1.000	-1958.3596	586.4729
	ST3A2	-594.6933	326.28188	1.000	-1867.1096	677.7229
	ST3H6	-2393.8333*	326.28188	.000	-3666.2496	-1121.4171
	Tru	-3048.5967*	326.28188	.000	-4321.0129	-1776.1804
Rno	A5	929.2833	326.28188	.742	-343.1329	2201.6996
	B1	686.5967	326.28188	1.000	-585.8196	1959.0129
	Ctrl	675.0333	326.28188	1.000	-597.3829	1947.4496
	Dre	466.5133	326.28188	1.000	-805.9029	1738.9296
	G1	505.9533	326.28188	1.000	-766.4629	1778.3696
	Gac	60.7700	326.28188	1.000	-1211.6462	1333.1862
	Gga	1115.3533	326.28188	.177	-157.0629	2387.7696
	Neg	2927.5800*	326.28188	.000	1655.1638	4199.9962
	ST2	2273.0633*	326.28188	.000	1000.6471	3545.4796
	ST3A1	2241.6367*	326.28188	.000	969.2204	3514.0529
	ST3A2	2332.8867*	326.28188	.000	1060.4704	3605.3029
	ST3H6	533.7467	326.28188	1.000	-738.6696	1806.1629
	Tru	-121.0167	326.28188	1.000	-1393.4329	1151.3996
ST2	A5	-1343.7800*	326.28188	.028	-2616.1962	-71.3638
	B1	-1586.4667*	326.28188	.004	-2858.8829	-314.0504
	Ctrl	-1598.0300*	326.28188	.003	-2870.4462	-325.6138
	Dre	-1806.5500*	326.28188	.001	-3078.9662	-534.1338
	G1	-1767.1100*	326.28188	.001	-3039.5262	-494.6938
	Gac	-2212.2933*	326.28188	.000	-3484.7096	-939.8771
	Gga	-1157.7100	326.28188	.127	-2430.1262	114.7062
	Neg	654.5167	326.28188	1.000	-617.8996	1926.9329
	Rno	-2273.0633*	326.28188	.000	-3545.4796	-1000.6471
	ST3A1	-31.4267	326.28188	1.000	-1303.8429	1240.9896
	ST3A2	59.8233	326.28188	1.000	-1212.5929	1332.2396
	ST3H6	-1739.3167*	326.28188	.001	-3011.7329	-466.9004
	Tru	-2394.0800*	326.28188	.000	-3666.4962	-1121.6638
ST3A1	A5	-1312.3533*	326.28188	.036	-2584.7696	-39.9371
	B1	-1555.0400*	326.28188	.005	-2827.4562	-282.6238
	Ctrl	-1566.6033*	326.28188	.004	-2839.0196	-294.1871
	Dre	-1775.1233*	326.28188	.001	-3047.5396	-502.7071
	G1	-1735.6833*	326.28188	.001	-3008.0996	-463.2671
	Gac	-2180.8667*	326.28188	.000	-3453.2829	-908.4504
	Gga	-1126.2833	326.28188	.163	-2398.6996	146.1329
	Neg	685.9433	326.28188	1.000	-586.4729	1958.3596
	Rno	-2241.6367*	326.28188	.000	-3514.0529	-969.2204

	ST2	31.4267	326.28188	1.000	-1240.9896	1303.8429
	ST3A2	91.2500	326.28188	1.000	-1181.1662	1363.6662
	ST3H6	-1707.8900*	326.28188	.001	-2980.3062	-435.4738
	Tru	-2362.6533*	326.28188	.000	-3635.0696	-1090.2371
ST3A2	A5	-1403.6033*	326.28188	.017	-2676.0196	-131.1871
	B1	-1646.2900*	326.28188	.002	-2918.7062	-373.8738
	Ctrl	-1657.8533*	326.28188	.002	-2930.2696	-385.4371
	Dre	-1866.3733*	326.28188	.000	-3138.7896	-593.9571
	G1	-1826.9333*	326.28188	.000	-3099.3496	-554.5171
	Gac	-2272.1167*	326.28188	.000	-3544.5329	-999.7004
	Gga	-1217.5333	326.28188	.078	-2489.9496	54.8829
	Neg	594.6933	326.28188	1.000	-677.7229	1867.1096
	Rno	-2332.8867*	326.28188	.000	-3605.3029	-1060.4704
	ST2	-59.8233	326.28188	1.000	-1332.2396	1212.5929
	ST3A1	-91.2500	326.28188	1.000	-1363.6662	1181.1662
	ST3H6	-1799.1400*	326.28188	.001	-3071.5562	-526.7238
	Tru	-2453.9033*	326.28188	.000	-3726.3196	-1181.4871
ST3H6	A5	395.5367	326.28188	1.000	-876.8796	1667.9529
	B1	152.8500	326.28188	1.000	-1119.5662	1425.2662
	Ctrl	141.2867	326.28188	1.000	-1131.1296	1413.7029
	Dre	-67.2333	326.28188	1.000	-1339.6496	1205.1829
	G1	-27.7933	326.28188	1.000	-1300.2096	1244.6229
	Gac	-472.9767	326.28188	1.000	-1745.3929	799.4396
	Gga	581.6067	326.28188	1.000	-690.8096	1854.0229
	Neg	2393.8333*	326.28188	.000	1121.4171	3666.2496
	Rno	-533.7467	326.28188	1.000	-1806.1629	738.6696
	ST2	1739.3167*	326.28188	.001	466.9004	3011.7329
	ST3A1	1707.8900*	326.28188	.001	435.4738	2980.3062
	ST3A2	1799.1400*	326.28188	.001	526.7238	3071.5562
	Tru	-654.7633	326.28188	1.000	-1927.1796	617.6529
Tru	A5	1050.3000	326.28188	.295	-222.1162	2322.7162
	B1	807.6133	326.28188	1.000	-464.8029	2080.0296
	Ctrl	796.0500	326.28188	1.000	-476.3662	2068.4662
	Dre	587.5300	326.28188	1.000	-684.8862	1859.9462
	G1	626.9700	326.28188	1.000	-645.4462	1899.3862
	Gac	181.7867	326.28188	1.000	-1090.6296	1454.2029
	Gga	1236.3700	326.28188	.067	-36.0462	2508.7862
	Neg	3048.5967*	326.28188	.000	1776.1804	4321.0129
	Rno	121.0167	326.28188	1.000	-1151.3996	1393.4329
	ST2	2394.0800*	326.28188	.000	1121.6638	3666.4962

ST3A1	2362.6533*	326.28188	.000	1090.2371	3635.0696
ST3A2	2453.9033*	326.28188	.000	1181.4871	3726.3196
ST3H6	654.7633	326.28188	1.000	-617.6529	1927.1796

Based on observed means.

The error term is Mean Square(Error) = 159689.794.

*. The mean difference is significant at the .05 level.

Profile Plots

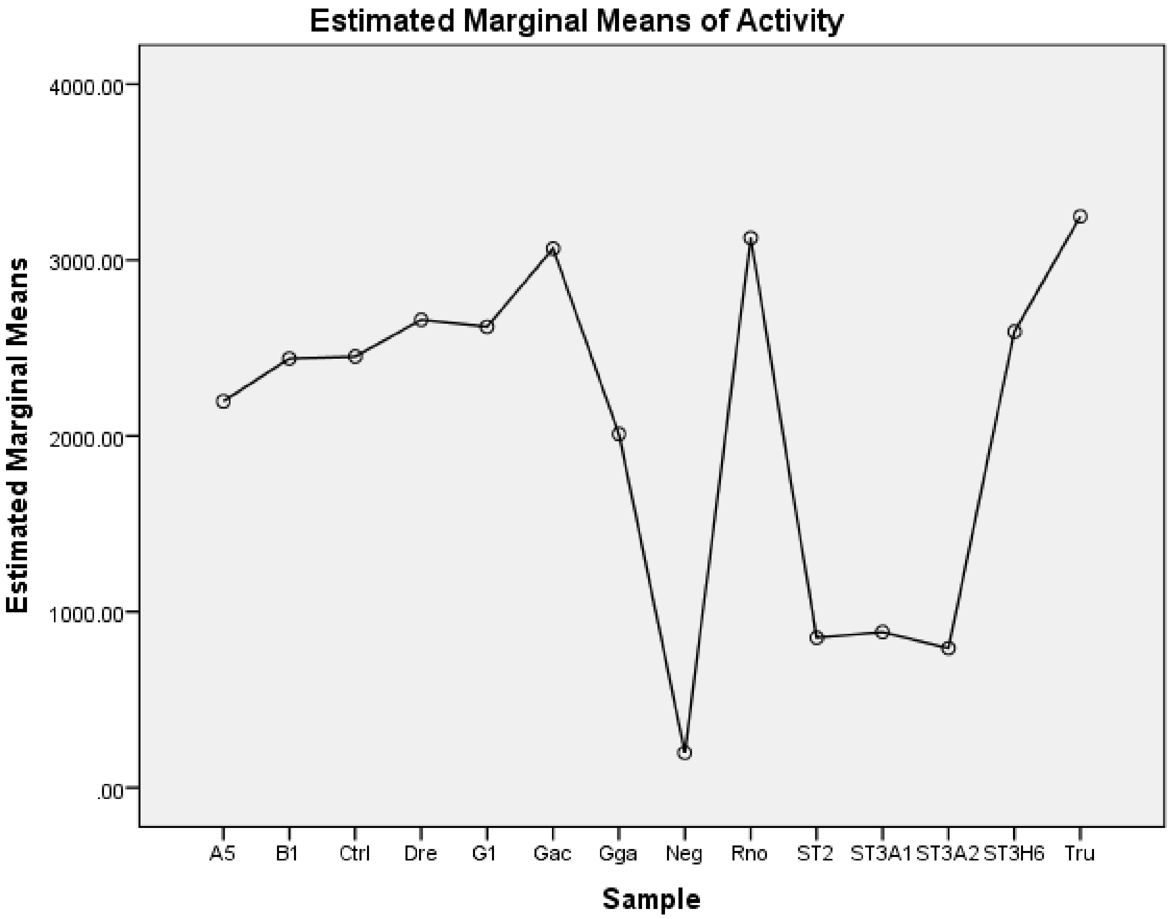

Supplement: Supplemental Information 10 — Proteins purified on Ni Sepharose excel (150 ng) were assayed using LacNAc (2.4 mM) as acceptor and CMP-Neu5Ac (0.2 mM) as donor. Reactions were incubated for 30 min at 37 ° C. Transfer of Neu5Ac was measured by release of phosphate using a phosphate linked assay with Malachite Green detection. The averages of three separate experiments are shown in Figure 3. Statistical analysis–part A. See Table 1 for explanation of all clone names. [file peerj-07-5788-s010.pdf]
