# Supplementary material for: Engineering of CHO cells for the production of vertebrate recombinant sialyltransferases
Source: PeerJ. 2019 Feb 11;7:e5788. doi: 10.7717/peerj.5788 (PMC6375257; doi:10.7717/peerj.5788)
Supplement: Supplemental Information 11 — Proteins purified on Ni Sepharose excel (150 ng) were assayed using LacNAc (2.4 mM) as acceptor and CMP-Neu5Ac (0.2 mM) as donor. Reactions were incubated for 30 min at 37 ° C. Transfer of Neu5Ac was measured by release of phosphate using a phosphate linked assay with Malachite Green detection. The averages of three separate experiments are shown in Figure 3. Statistical analysis–part B. See Table 1 for explanation of all clone names. [file peerj-07-5788-s011.pdf]

Figure 3: Statistics

```
GET
  FILE='\\gweng.gmadtree.gmit.ie\STAFF\Benoit.Houeix\Documents\ST.sav'.
DATASET NAME DataSet1 WINDOW=FRONT.
T-TEST GROUPS=Sample('A5' 'Ctrl')
  /MISSING=ANALYSIS
  /VARIABLES=Activity
  /CRITERIA=CI(.95).
```

Legend to Figure 3 Raw Data Set 3

Label for Statistics	Corresponding in Manuscript
A5	hST6 A5
B1	hST6 B1
G1	hST6
Dre	zST6
Gac	sST6
Tru	fST6
Gga	cST6
Rno	rST6
Ctrl	mST6 (commercial mouse enzyme)
ST3A2	hST3 A2
ST3A1	hST3 A2
ST3H6	zST3
ST2	hST6Gal2
Neg	Neg (IMAC-purified PSA[1])

[1]PSA: Prostate Specific Antigen (no sialyltransferase activity)

T-Test

Note: These means are pmol/well, NOT pmol/min/µg protein as presented in Figure 3.

Notes

Output Created		12-JUL-2018 10:15:41
Comments		
Input	Data	\\gweng.gmadtree.gmit.ie\STAFF\Benoit.Houeix\Documents\ST.sav
	Active Dataset	DataSet1
	Filter	<none>

	Weight	<none>
	Split File	<none>
	N of Rows in Working Data File	48
Missing Value Handling	Definition of Missing	User defined missing values are treated as missing.
	Cases Used	Statistics for each analysis are based on the cases with no missing or out-of-range data for any variable in the analysis.
Syntax		T-TEST GROUPS=Sample('A5' 'Ctrl') /MISSING=ANALYSIS /VARIABLES=Activity /CRITERIA=CI(.95).
Resources	Processor Time	00:00:00.00
	Elapsed Time	00:00:00.00

```
[DataSet1] \\gweng.gmadtree.gmit.ie\STAFF\Benoit.Houeix\Documents\ST.sav
```

Group Statistics

	Sample	N	Mean	Std. Deviation	Std. Error Mean
Activity	A5	3	2197.3667	312.12468	180.20527
	Ctrl	3	2451.6167	455.31970	262.87895

Independent Samples Test

		Levene's Test for Equality of Variances		t-test for Equality of Means	
		F	Sig.	t	df
Activity	Equal variances assumed	.570	.492	-.798	4
	Equal variances not assumed			-.798	3.540

Independent Samples Test

	t-test for Equality of Means

		Sig. (2-tailed)	Mean Difference	Std. Error Difference	95% Confidence Interval of the Difference
					Lower
Activity	Equal variances assumed	.470	-254.25000	318.71505	-1139.14484
	Equal variances not assumed	.475	-254.25000	318.71505	-1186.43005

Independent Samples Test

		t-test for Equality of Means
		95% Confidence Interval of the Difference
		Upper
Activity	Equal variances assumed	630.64484
	Equal variances not assumed	677.93005

```
T-TEST GROUPS=Sample('B1' 'Ctrl')
  /MISSING=ANALYSIS
  /VARIABLES=Activity
  /CRITERIA=CI(.95).
```

T-Test

Notes

Output Created		12-JUL-2018 10:17:22
Comments		
Input	Data	\\gweng.gmadtree.gmit.ie\STAFF\Benoit.Houeix\Documents\ST.sav
	Active Dataset	DataSet1
	Filter	<none>
	Weight	<none>
	Split File	<none>
	N of Rows in Working Data File	48
Missing Value Handling	Definition of Missing	User defined missing values are treated as missing.

	Cases Used	Statistics for each analysis are based on the cases with no missing or out-of-range data for any variable in the analysis.
Syntax		T-TEST GROUPS=Sample('B1' 'Ctrl') /MISSING=ANALYSIS /VARIABLES=Activity /CRITERIA=CI(.95).
Resources	Processor Time	00:00:00.00
	Elapsed Time	00:00:00.00

Group Statistics

	Sample	N	Mean	Std. Deviation	Std. Error Mean
Activity	B1	3	2440.0533	584.34650	337.37261
	Ctrl	3	2451.6167	455.31970	262.87895

Independent Samples Test

		Levene's Test for Equality of Variances		t-test for Equality of Means	
		F	Sig.	t	df
Activity	Equal variances assumed	.098	.769	-.027	4
	Equal variances not assumed			-.027	3.774

Independent Samples Test

		t-test for Equality of Means			
					95% Confidence Interval of the Difference
		Sig. (2-tailed)	Mean Difference	Std. Error Difference	Lower
Activity	Equal variances assumed	.980	-11.56333	427.69805	-1199.04349
	Equal variances not assumed	.980	-11.56333	427.69805	-1227.55702

Independent Samples Test

	t-test for Equality of Means

		95% Confidence Interval of the Difference
		Upper
Activity	Equal variances assumed	1175.91682
	Equal variances not assumed	1204.43036

```
T-TEST GROUPS=Sample('G1' 'Ctrl')
  /MISSING=ANALYSIS
  /VARIABLES=Activity
  /CRITERIA=CI(.95).
```

T-Test

Notes

Output Created		12-JUL-2018 10:19:44
Comments		
Input	Data	\\gweng.gmadtree.gmit.ie\STAFF\Benoit.Houeix\Documents\ST.sav
	Active Dataset	DataSet1
	Filter	<none>
	Weight	<none>
	Split File	<none>
	N of Rows in Working Data File	48
Missing Value Handling	Definition of Missing	User defined missing values are treated as missing.
	Cases Used	Statistics for each analysis are based on the cases with no missing or out-of-range data for any variable in the analysis.
Syntax		T-TEST GROUPS=Sample('G1' 'Ctrl') /MISSING=ANALYSIS /VARIABLES=Activity /CRITERIA=CI(.95).
Resources	Processor Time	00:00:00.00
	Elapsed Time	00:00:00.02

Group Statistics

	Sample	N	Mean	Std. Deviation	Std. Error Mean
Activity	G1	3	2620.6967	345.92251	199.71846
	Ctrl	3	2451.6167	455.31970	262.87895

Independent Samples Test

		Levene's Test for Equality of Variances		t-test for Equality of Means	
		F	Sig.	t	df
Activity	Equal variances assumed	.288	.620	.512	4
	Equal variances not assumed			.512	3.732

Independent Samples Test

		t-test for Equality of Means			
		Sig. (2-tailed)	Mean Difference	Std. Error Difference	95% Confidence Interval of the Difference
					Lower
Activity	Equal variances assumed	.636	169.08000	330.14058	-747.53721
	Equal variances not assumed	.637	169.08000	330.14058	-774.10883

Independent Samples Test

		t-test for Equality of Means
		95% Confidence Interval of the Difference
		Upper
Activity	Equal variances assumed	1085.69721
	Equal variances not assumed	1112.26883

```
T-TEST GROUPS=Sample('Dre' 'Ctrl')
  /MISSING=ANALYSIS
  /VARIABLES=Activity
  /CRITERIA=CI(.95).
```

T-Test

Notes

Output Created		12-JUL-2018 10:20:10
Comments		
Input	Data	\\gweng.gmadtree.gmit.ie\STAFF\Benoit.Houeix\Documents\ST.sav
	Active Dataset	DataSet1
	Filter	<none>
	Weight	<none>

	Split File	<none>
	N of Rows in Working Data File	48
Missing Value Handling	Definition of Missing	User defined missing values are treated as missing.
	Cases Used	Statistics for each analysis are based on the cases with no missing or out-of-range data for any variable in the analysis.
Syntax		T-TEST GROUPS=Sample('Dre' 'Ctrl')<br>/MISSING=ANALYSIS<br>/VARIABLES=Activity<br>/CRITERIA=CI(.95).
Resources	Processor Time	00:00:00.03
	Elapsed Time	00:00:00.02

Group Statistics

	Sample	N	Mean	Std. Deviation	Std. Error Mean
Activity	Dre	3	2660.1367	397.39347	229.43523
	Ctrl	3	2451.6167	455.31970	262.87895

Independent Samples Test

		Levene's Test for Equality of Variances		t-test for Equality of Means	
		F	Sig.	t	df
Activity	Equal variances assumed	.065	.811	.598	4
	Equal variances not assumed			.598	3.928

Independent Samples Test

		t-test for Equality of Means			
					95% Confidence Interval of the Difference
		Sig. (2-tailed)	Mean Difference	Std. Error Difference	Lower
Activity	Equal variances assumed	.582	208.52000	348.92100	-760.24002
	Equal variances not assumed	.583	208.52000	348.92100	-767.26792

Independent Samples Test

		t-test for Equality of Means
		95% Confidence Interval of the Difference
		Upper
Activity	Equal variances assumed	1177.28002
	Equal variances not assumed	1184.30792

```
T-TEST GROUPS=Sample('Gac' 'Ctrl')
  /MISSING=ANALYSIS
  /VARIABLES=Activity
  /CRITERIA=CI(.95).
```

T-Test

Notes

Output Created		12-JUL-2018 10:20:28
Comments		
Input	Data	\\gweng.gmadtree.gmit.ie\STAFF\Benoit.Houeix\Documents\ST.sav
	Active Dataset	DataSet1
	Filter	<none>
	Weight	<none>
	Split File	<none>
	N of Rows in Working Data File	48
Missing Value Handling	Definition of Missing	User defined missing values are treated as missing.
	Cases Used	Statistics for each analysis are based on the cases with no missing or out-of-range data for any variable in the analysis.
Syntax		T-TEST GROUPS=Sample('Gac' 'Ctrl') /MISSING=ANALYSIS /VARIABLES=Activity /CRITERIA=CI(.95).
Resources	Processor Time	00:00:00.00
	Elapsed Time	00:00:00.02

Group Statistics

	Sample	N	Mean	Std. Deviation	Std. Error Mean
Activity	Gac	3	3065.8800	432.52984	249.72122
	Ctrl	3	2451.6167	455.31970	262.87895

Independent Samples Test

		Levene's Test for Equality of Variances		t-test for Equality of Means	
		F	Sig.	t	df
Activity	Equal variances assumed	.001	.982	1.694	4
	Equal variances not assumed			1.694	3.989

Independent Samples Test

		t-test for Equality of Means			
		Sig. (2-tailed)	Mean Difference	Std. Error Difference	95% Confidence Interval of the Difference Lower
Activity	Equal variances assumed	.165	614.26333	362.58245	-392.42693
	Equal variances not assumed	.166	614.26333	362.58245	-393.47276

Independent Samples Test

		t-test for Equality of Means 95% Confidence Interval of the Difference
		Upper
Activity	Equal variances assumed	1620.95360
	Equal variances not assumed	1621.99943

```
T-TEST GROUPS=Sample('Tru' 'Ctrl')
  /MISSING=ANALYSIS
  /VARIABLES=Activity
  /CRITERIA=CI(.95).
```

T-Test

Notes

Output Created		12-JUL-2018 10:20:41
Comments		
Input	Data	\\gweng.gmadtree.gmit.ie\STAFF\Benoit.Houeix\Documents\ST.sav
	Active Dataset	DataSet1
	Filter	<none>
	Weight	<none>
	Split File	<none>

	N of Rows in Working Data File	48
Missing Value Handling	Definition of Missing	User defined missing values are treated as missing.
	Cases Used	Statistics for each analysis are based on the cases with no missing or out-of-range data for any variable in the analysis.
Syntax		T-TEST GROUPS=Sample('Tru' 'Ctrl')<br>/MISSING=ANALYSIS<br>/VARIABLES=Activity<br>/CRITERIA=CI(.95).
Resources	Processor Time	00:00:00.00
	Elapsed Time	00:00:00.02

Group Statistics

	Sample	N	Mean	Std. Deviation	Std. Error Mean
Activity	Tru	3	3247.6667	434.25077	250.71480
	Ctrl	3	2451.6167	455.31970	262.87895

Independent Samples Test

		Levene's Test for Equality of Variances		t-test for Equality of Means	
		F	Sig.	t	df
Activity	Equal variances assumed	.001	.978	2.191	4
	Equal variances not assumed			2.191	3.991

Independent Samples Test

		t-test for Equality of Means			
					95% Confidence Interval of the Difference
		Sig. (2-tailed)	Mean Difference	Std. Error Difference	Lower
Activity	Equal variances assumed	.094	796.05000	363.26747	-212.54218
	Equal variances not assumed	.094	796.05000	363.26747	-213.43432

Independent Samples Test

	t-test for Equality of Means

		95% Confidence Interval of the Difference
		Upper
Activity	Equal variances assumed	1804.64218
	Equal variances not assumed	1805.53432

```
T-TEST GROUPS=Sample('Gga' 'Ctrl')
  /MISSING=ANALYSIS
  /VARIABLES=Activity
  /CRITERIA=CI(.95).
```

T-Test

Notes

Output Created		12-JUL-2018 10:20:58
Comments		
Input	Data	\\gweng.gmadtree.gmit.ie\STAFF\Benoit.Houeix\Documents\ST.sav
	Active Dataset	DataSet1
	Filter	<none>
	Weight	<none>
	Split File	<none>
	N of Rows in Working Data File	48
Missing Value Handling	Definition of Missing	User defined missing values are treated as missing.
	Cases Used	Statistics for each analysis are based on the cases with no missing or out-of-range data for any variable in the analysis.
Syntax		T-TEST GROUPS=Sample('Gga' 'Ctrl') /MISSING=ANALYSIS /VARIABLES=Activity /CRITERIA=CI(.95).
Resources	Processor Time	00:00:00.00
	Elapsed Time	00:00:00.00

Group Statistics

	Sample	N	Mean	Std. Deviation	Std. Error Mean
Activity	Gga	3	2011.2967	280.50808	161.95141
	Ctrl	3	2451.6167	455.31970	262.87895

Independent Samples Test

		Levene's Test for Equality of Variances		t-test for Equality of Means	
		F	Sig.	t	df
Activity	Equal variances assumed	.936	.388	-1.426	4
	Equal variances not assumed			-1.426	3.327

Independent Samples Test

		t-test for Equality of Means			
					95% Confidence Interval of the Difference
		Sig. (2-tailed)	Mean Difference	Std. Error Difference	Lower
Activity	Equal variances assumed	.227	-440.32000	308.76140	-1297.57909
	Equal variances not assumed	.241	-440.32000	308.76140	-1370.49606

Independent Samples Test

		t-test for Equality of Means
		95% Confidence Interval of the Difference
		Upper
Activity	Equal variances assumed	416.93909
	Equal variances not assumed	489.85606

```
T-TEST GROUPS=Sample('Rno' 'Ctrl')
  /MISSING=ANALYSIS
  /VARIABLES=Activity
  /CRITERIA=CI(.95).
```

T-Test

Notes

Output Created		12-JUL-2018 10:21:16
Comments		
Input	Data	\\gweng.gmadtree.gmit.ie\STAFF\Benoit.Houeix\Documents\ST.sav
	Active Dataset	DataSet1
	Filter	<none>
	Weight	<none>
	Split File	<none>

	N of Rows in Working Data File	48
Missing Value Handling	Definition of Missing	User defined missing values are treated as missing.
	Cases Used	Statistics for each analysis are based on the cases with no missing or out-of-range data for any variable in the analysis.
Syntax		T-TEST GROUPS=Sample('Rno' 'Ctrl') /MISSING=ANALYSIS /VARIABLES=Activity /CRITERIA=CI(.95).
Resources	Processor Time	00:00:00.03
	Elapsed Time	00:00:00.02

Group Statistics

	Sample	N	Mean	Std. Deviation	Std. Error Mean
Activity	Rno	3	3126.6500	659.51091	380.76880
	Ctrl	3	2451.6167	455.31970	262.87895

Independent Samples Test

		Levene's Test for Equality of Variances		t-test for Equality of Means	
		F	Sig.	t	df
Activity	Equal variances assumed	.391	.566	1.459	4
	Equal variances not assumed			1.459	3.554

Independent Samples Test

		t-test for Equality of Means			
		Sig. (2-tailed)	Mean Difference	Std. Error Difference	95% Confidence Interval of the Difference Lower
Activity	Equal variances assumed	.218	675.03333	462.69885	-609.62462
	Equal variances not assumed	.227	675.03333	462.69885	-675.84150

Independent Samples Test

	t-test for Equality of Means

		95% Confidence Interval of the Difference
		Upper
Activity	Equal variances assumed	1959.69129
	Equal variances not assumed	2025.90817
