# Supplementary material for: Engineering of CHO cells for the production of vertebrate recombinant sialyltransferases
Source: PeerJ. 2019 Feb 11;7:e5788. doi: 10.7717/peerj.5788 (PMC6375257; doi:10.7717/peerj.5788)
Supplement: Supplemental Information 12 — Various ST6Gal I and ST3Gal IV purified proteins on Western blots detected by mouse anti-His, HRP-goat anti-mouse IgG with DAB staining. PNGase F digested ST6Gal I (+) and undigested (−) control. Loading was ~2ug purified from ProCHO-AT media separated on 12 % NuPAGE Novex Bis-Tris gels in MOPS buffer under reducing conditions. Marker: BenchMark His-tagged protein standard. See Table 1 for explanation of all clone names. [file peerj-07-5788-s012.pdf]

Figure 4A – part 1

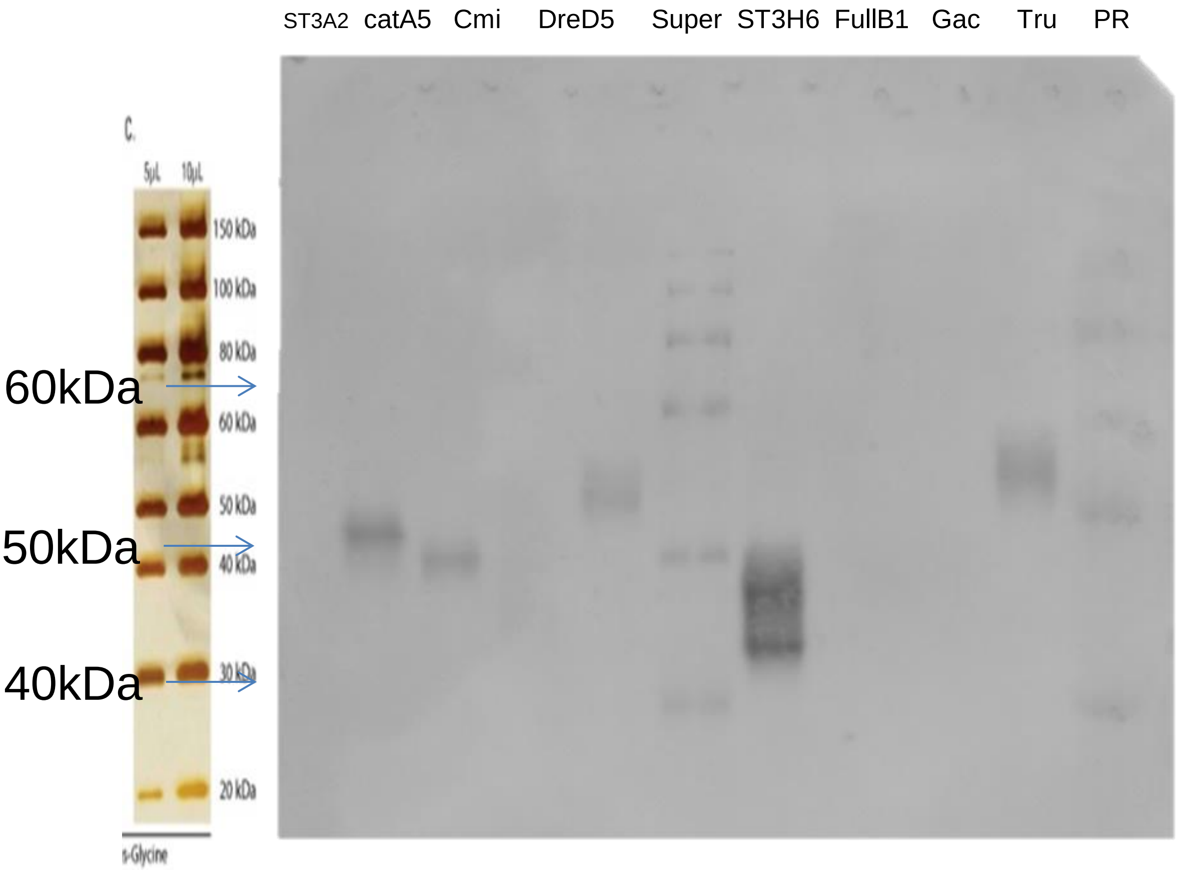

Figure 4A: Uses 6 lanes from this gel
Lane 1 of Fig 4A: See Lane 1 this gel (“ST3A2”)
Lane 2 of Fig 4A: See Lane 5 this gel (“Super”)
Lane 3 of Fig 4A: See Lane 6 this gel (“ST3H6”)
Lane 4 of Fig 4A: See Lane 2 this gel (“catA5”)
Lane 5 of Fig 4A: See Lane 4 this gel (“DreD5”)
Lane 6 of Fig 4A: See Lane 9 this gel (“Tru”)
Lane 7 & 8 of Fig 4A: part 2

Figure 4A: part 2

A1 Rno Cd73 NP SS TP TC Lys1 Lys2 Lys3

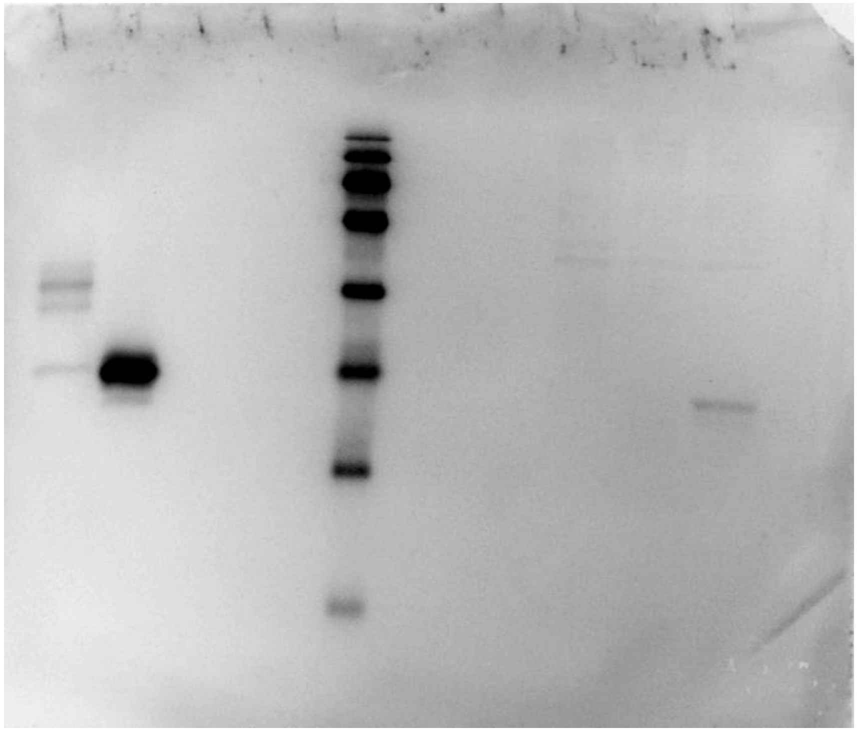

SuperSignal ladder, Thermo

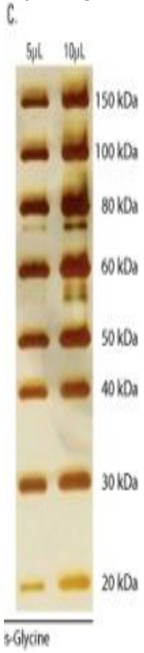

Figure 4A: Uses 2 lanes from this gel
Lane 1-6 of Fig 4A: See part 1
Lane 7 of Fig 4A: See Lane 1 this gel (“A1”)
Lane 8 of Fig 4A: See Lane 2 this gel (“Rno”)
Markers based on Lane 5 (“SS”)

12% SDS-PAGE – 150V for 2h – WB Sigma antiHis-HRP 1/1000 1h rt – Advansta detection

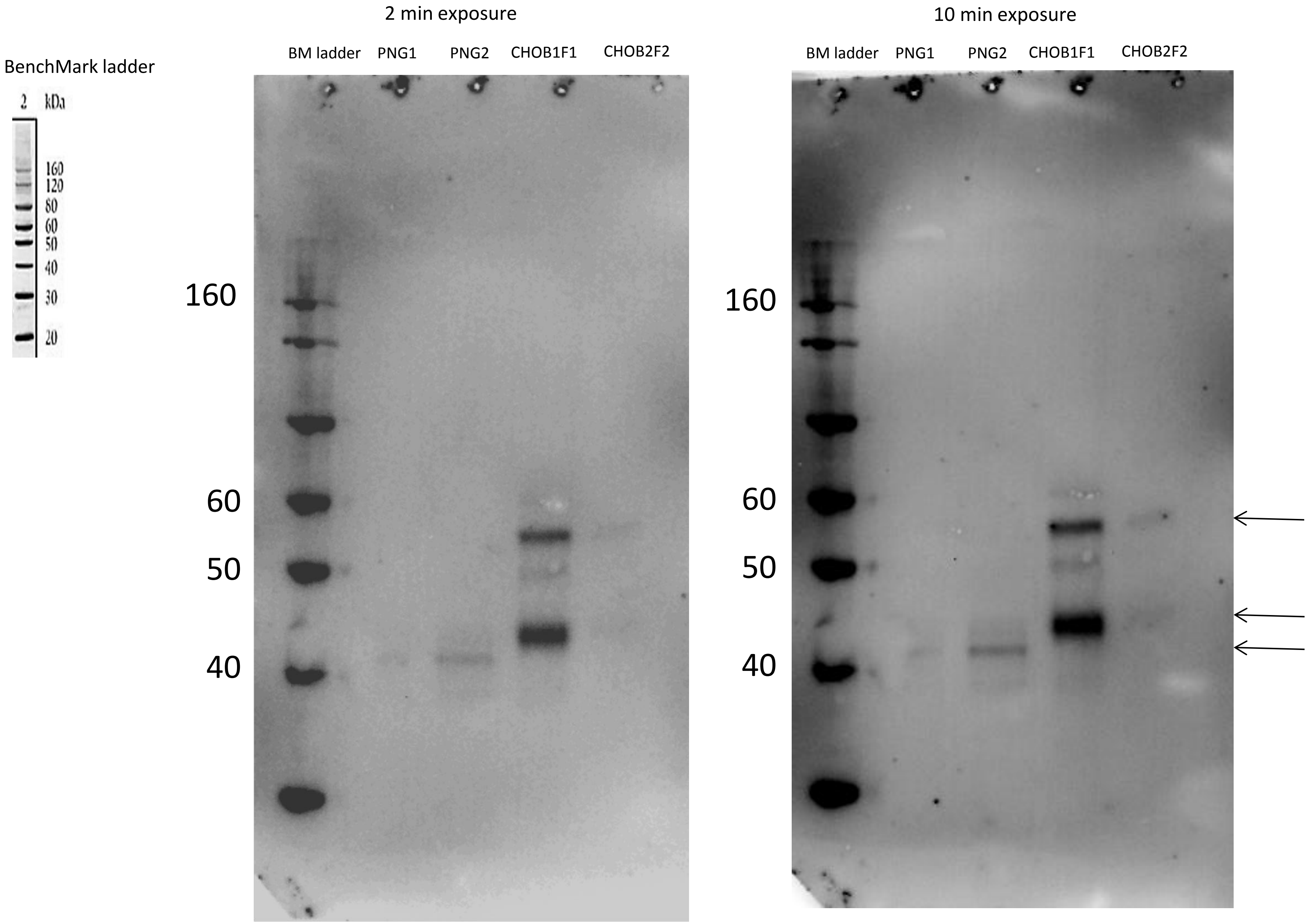

This is the original for Figure 4B. Two lanes will have been cut from this for the final figure.

Figure 4B: Uses 2 lanes from this gel (Slide 3)
Lane + of Fig 4B: See Lane 3 this gel (10 min, “PNG2”)
Lane - of Fig 4B: See Lane 4 this gel (10 min “CHOB1F1”)
Markers for Fig 4B: See Lane 1 this gel (10 min “BM ladder”)
