# Supplementary material for: Engineering of CHO cells for the production of vertebrate recombinant sialyltransferases
Source: PeerJ. 2019 Feb 11;7:e5788. doi: 10.7717/peerj.5788 (PMC6375257; doi:10.7717/peerj.5788)
Supplement: Supplemental Information 13 — ASF, reacted with each of the sialyltransferase preparations (37 ° C, 16 h), was bound to biotinylated SNA-I lectin. Binding to Neu5Ac on the ASF was detected with HRP-streptavidin. Expt 1 (black bars–Figure 6A) included ST6Gal I, ST3Gal IV and ST6Gal II preparations. Expt 2 (grey bars–Figure 6A) included only ST6Gal I preparations. Each sample was analysed for binding with biotinylated RCA I lectin (Figure 6B). All values are relative to ASF. Each sample was assayed in triplicate. Statistics–part A (SNA-I t-tests). [file peerj-07-5788-s013.pdf]

Figure 6:T-Test

Legend to Figure 6 Raw Data Set 1

Label for Statistics	Corresponding in Manuscript
ASF	ASF (Asialofetuin)
A5	hST6 A5
B1	hST6 B1
G1	hST6
Dre	zST6
Gac	sST6
Tru	fST6
Gga	cST6
Rno	rST6
Ctrl	mST6 (commercial mouse enzyme)
ST3A2	hST3 A2
ST3A1	hST3 A2
ST3H6	zST3
ST2	hST6Gal2

T-Test: hST6 A5

Notes

Output Created		11-JUL-2018 17:09:44
Comments		
Input	Data	\\gweng.gmadtree.gmit.ie\STAFF\Benoit.Houeix\Documents\ST.sav
	Active Dataset	DataSet1
	Filter	<none>
	Weight	<none>
	Split File	<none>
	N of Rows in Working Data File	45
Missing Value Handling	Definition of Missing	User defined missing values are treated as missing.
	Cases Used	Statistics for each analysis are based on the cases with no missing or out-of-range data for any variable in the analysis.

Syntax		T-TEST GROUPS=Sample('A5' 'ASF')<br>/MISSING=ANALYSIS<br>/VARIABLES=value1<br>/CRITERIA=CI(.95).
Resources	Processor Time	00:00:00.00
	Elapsed Time	00:00:00.03

Group Statistics

	Sample	N	Mean	Std. Deviation	Std. Error Mean
SNA-I	A5	3	.77287	.045671	.026368
	ASF	3	.60230	.008643	.004990

Independent Samples Test

		Levene's Test for Equality of Variances		t-test for Equality of Means	
		F	Sig.	t	df
SNA-I	Equal variances assumed	3.018	.157	6.356	4
	Equal variances not assumed			6.356	2.143

Independent Samples Test

		t-test for Equality of Means			
					95% Confidence Interval of the Difference
		Sig. (2-tailed)	Mean Difference	Std. Error Difference	Lower
SNA-I	Equal variances assumed	.003	.170567	.026836	.096058
	Equal variances not assumed	.020	.170567	.026836	.062183

Independent Samples Test

		t-test for Equality of Means
		95% Confidence Interval of the Difference
		Upper
SNA-I	Equal variances assumed	.245076
	Equal variances not assumed	.278951

Notes

Output Created		11-JUL-2018 17:09:57
Comments		
Input	Data	\\gweng.gmadtree.gmit.ie\STAFF\Benoit.Houeix\Documents\ST.sav
	Active Dataset	DataSet1
	Filter	<none>
	Weight	<none>
	Split File	<none>
	N of Rows in Working Data File	45
Missing Value Handling	Definition of Missing	User defined missing values are treated as missing.
	Cases Used	Statistics for each analysis are based on the cases with no missing or out-of-range data for any variable in the analysis.
Syntax		T-TEST GROUPS=Sample('1' 'ASF')<br>/MISSING=ANALYSIS<br>/VARIABLES=value1<br>/CRITERIA=CI(.95).
Resources	Processor Time	00:00:00.00
	Elapsed Time	00:00:00.01

T-Test: hST6 B1

Notes

Output Created		11-JUL-2018 17:10:23
Comments		
Input	Data	\\gweng.gmadtree.gmit.ie\STAFF\Benoit.Houeix\Documents\ST.sav
	Active Dataset	DataSet1
	Filter	<none>
	Weight	<none>
	Split File	<none>
	N of Rows in Working Data File	45
Missing Value Handling	Definition of Missing	User defined missing values are treated as missing.
	Cases Used	Statistics for each analysis are based on the cases with no missing or out-of-range data for any variable in the analysis.
Syntax		T-TEST GROUPS=Sample('B1' 'ASF') /MISSING=ANALYSIS /VARIABLES=value1 /CRITERIA=CI(.95).
Resources	Processor Time	00:00:00.00
	Elapsed Time	00:00:00.05

Group Statistics

	Sample	N	Mean	Std. Deviation	Std. Error Mean
SNA-I	B1	3	1.38367	.074086	.042774
	ASF	3	.60230	.008643	.004990

Independent Samples Test

		Levene's Test for Equality of Variances		t-test for Equality of Means	
		F	Sig.	t	df
SNA-I	Equal variances assumed	10.726	.031	18.144	4
	Equal variances not assumed			18.144	2.054

Independent Samples Test

		t-test for Equality of Means			
					95% Confidence Interval of the Difference
		Sig. (2-tailed)	Mean Difference	Std. Error Difference	Lower
SNA-I	Equal variances assumed	.000	.781367	.043064	.661803
	Equal variances not assumed	.003	.781367	.043064	.600705

Independent Samples Test

		t-test for Equality of Means
		95% Confidence Interval of the Difference
		Upper
SNA-I	Equal variances assumed	.900931
	Equal variances not assumed	.962028

T-Test: hST6

Notes

Output Created		11-JUL-2018 17:10:44
Comments		
Input	Data	\\gweng.gmadtree.gmit.ie\STAFF\Benoit.Houeix\Documents\ST.sav
	Active Dataset	DataSet1
	Filter	<none>
	Weight	<none>
	Split File	<none>
	N of Rows in Working Data File	45
Missing Value Handling	Definition of Missing	User defined missing values are treated as missing.
	Cases Used	Statistics for each analysis are based on the cases with no missing or out-of-range data for any variable in the analysis.
Syntax		T-TEST GROUPS=Sample('G1' 'ASF')<br>/MISSING=ANALYSIS<br>/VARIABLES=value1<br>/CRITERIA=CI(.95).

Resources	Processor Time	00:00:00.02
	Elapsed Time	00:00:00.02

Group Statistics

	Sample	N	Mean	Std. Deviation	Std. Error Mean
SNA-I	G1	3	.65920	.041122	.023742
	ASF	3	.60230	.008643	.004990

Independent Samples Test

		Levene's Test for Equality of Variances		t-test for Equality of Means	
		F	Sig.	t	df
SNA-I	Equal variances assumed	4.946	.090	2.345	4
	Equal variances not assumed			2.345	2.176

Independent Samples Test

		t-test for Equality of Means			
					95% Confidence Interval of the Difference
		Sig. (2-tailed)	Mean Difference	Std. Error Difference	Lower
SNA-I	Equal variances assumed	.079	.056900	.024261	-.010459
	Equal variances not assumed	.133	.056900	.024261	-.039784

Independent Samples Test

		t-test for Equality of Means
		95% Confidence Interval of the Difference
		Upper
SNA-I	Equal variances assumed	.124259
	Equal variances not assumed	.153584

T-Test: zST6

Notes

Output Created		11-JUL-2018 17:11:01
Comments		
Input	Data	\\gweng.gmadtree.gmit.ie\STAFF\Benoit.Houeix\Documents\ST.sav
	Active Dataset	DataSet1
	Filter	<none>
	Weight	<none>
	Split File	<none>
	N of Rows in Working Data File	45
Missing Value Handling	Definition of Missing	User defined missing values are treated as missing.
	Cases Used	Statistics for each analysis are based on the cases with no missing or out-of-range data for any variable in the analysis.
Syntax		T-TEST GROUPS=Sample('Dre' 'ASF') /MISSING=ANALYSIS /VARIABLES=value1 /CRITERIA=CI(.95).
Resources	Processor Time	00:00:00.00
	Elapsed Time	00:00:00.00

Group Statistics

	Sample	N	Mean	Std. Deviation	Std. Error Mean
SNA-I	Dre	3	1.07970	.057551	.033227
	ASF	3	.60230	.008643	.004990

Independent Samples Test

		Levene's Test for Equality of Variances		t-test for Equality of Means	
		F	Sig.	t	df
SNA-I	Equal variances assumed	9.166	.039	14.208	4

	Equal variances not assumed			14.208	2.090

Independent Samples Test

		t-test for Equality of Means			
					95% Confidence Interval of the Difference
		Sig. (2-tailed)	Mean Difference	Std. Error Difference	Lower
SNA-I	Equal variances assumed	.000	.477400	.033600	.384112
	Equal variances not assumed	.004	.477400	.033600	.338647

Independent Samples Test

		t-test for Equality of Means
		95% Confidence Interval of the Difference
		Upper
SNA-I	Equal variances assumed	.570688
	Equal variances not assumed	.616153

T-Test: sST6

Notes

Output Created		11-JUL-2018 17:11:19
Comments		
Input	Data	\\gweng.gmadtree.gmit.ie\STAFF\Benoit.Houeix\Documents\ST.sav
	Active Dataset	DataSet1
	Filter	<none>
	Weight	<none>
	Split File	<none>
	N of Rows in Working Data File	45
Missing Value Handling	Definition of Missing	User defined missing values are treated as missing.

	Cases Used	Statistics for each analysis are based on the cases with no missing or out-of-range data for any variable in the analysis.
Syntax		T-TEST GROUPS=Sample('Gac' 'ASF')<br>/MISSING=ANALYSIS<br>/VARIABLES=value1<br>/CRITERIA=CI(.95).
Resources	Processor Time	00:00:00.02
	Elapsed Time	00:00:00.03

Group Statistics

	Sample	N	Mean	Std. Deviation	Std. Error Mean
SNA-I	Gac	3	.81133	.140832	.081310
	ASF	3	.60230	.008643	.004990

Independent Samples Test

		Levene's Test for Equality of Variances		t-test for Equality of Means	
		F	Sig.	t	df
SNA-I	Equal variances assumed	13.858	.020	2.566	4
	Equal variances not assumed			2.566	2.015

Independent Samples Test

		t-test for Equality of Means			
					95% Confidence Interval of the Difference
		Sig. (2-tailed)	Mean Difference	Std. Error Difference	Lower
SNA-I	Equal variances assumed	.062	.209033	.081463	-.017143
	Equal variances not assumed	.123	.209033	.081463	-.138973

Independent Samples Test

	t-test for Equality of Means
	95% Confidence Interval of the Difference

		Upper
SNA-I	Equal variances assumed	.435210
	Equal variances not assumed	.557039

T-Test: fST6

Notes

Output Created		11-JUL-2018 17:11:41
Comments		
Input	Data	\\gweng.gmadtree.gmit.ie\STAFF\Benoit.Houeix\Documents\ST.sav
	Active Dataset	DataSet1
	Filter	<none>
	Weight	<none>
	Split File	<none>
	N of Rows in Working Data File	45
Missing Value Handling	Definition of Missing	User defined missing values are treated as missing.
	Cases Used	Statistics for each analysis are based on the cases with no missing or out-of-range data for any variable in the analysis.
Syntax		T-TEST GROUPS=Sample('Tru' 'ASF') /MISSING=ANALYSIS /VARIABLES=value1 /CRITERIA=CI(.95).
Resources	Processor Time	00:00:00.00
	Elapsed Time	00:00:00.02

Group Statistics

	Sample	N	Mean	Std. Deviation	Std. Error Mean
SNA-I	Tru	3	1.18953	.107552	.062095
	ASF	3	.60230	.008643	.004990

Independent Samples Test

		Levene's Test for Equality of Variances		t-test for Equality of Means	
		F	Sig.	t	df
SNA-I	Equal variances assumed	8.849	.041	9.427	4
	Equal variances not assumed			9.427	2.026

Independent Samples Test

		t-test for Equality of Means			
					95% Confidence Interval of the Difference
		Sig. (2-tailed)	Mean Difference	Std. Error Difference	Lower
SNA-I	Equal variances assumed	.001	.587233	.062295	.414274
	Equal variances not assumed	.011	.587233	.062295	.322447

Independent Samples Test

		t-test for Equality of Means
		95% Confidence Interval of the Difference
		Upper
SNA-I	Equal variances assumed	.760193
	Equal variances not assumed	.852020

T-Test: cST6

Notes

Output Created		11-JUL-2018 17:12:16
Comments		
Input	Data	\\gweng.gmadtree.gmit.ie\STAFF\Benoit.Houeix\Documents\ST.sav
	Active Dataset	DataSet1
	Filter	<none>
	Weight	<none>
	Split File	<none>
	N of Rows in Working Data File	45
Missing Value Handling	Definition of Missing	User defined missing values are treated as missing.

	Cases Used	Statistics for each analysis are based on the cases with no missing or out-of-range data for any variable in the analysis.
Syntax		T-TEST GROUPS=Sample('Gga' 'ASF')<br>/MISSING=ANALYSIS<br>/VARIABLES=value1<br>/CRITERIA=CI(.95).
Resources	Processor Time	00:00:00.00
	Elapsed Time	00:00:00.00

Group Statistics

	Sample	N	Mean	Std. Deviation	Std. Error Mean
SNA-I	Gga	3	2.55470	.110604	.063857
	ASF	3	.60230	.008643	.004990

Independent Samples Test

		Levene's Test for Equality of Variances		t-test for Equality of Means	
		F	Sig.	t	df
SNA-I	Equal variances assumed	6.556	.063	30.481	4
	Equal variances not assumed			30.481	2.024

Independent Samples Test

		t-test for Equality of Means			
					95% Confidence Interval of the Difference
		Sig. (2-tailed)	Mean Difference	Std. Error Difference	Lower
SNA-I	Equal variances assumed	.000	1.952400	.064052	1.774563
	Equal variances not assumed	.001	1.952400	.064052	1.679969

Independent Samples Test

		t-test for Equality of Means

		95% Confidence Interval of the Difference
		Upper
SNA-I	Equal variances assumed	2.130237
	Equal variances not assumed	2.224831

T-Test: rST6

Notes

Output Created		11-JUL-2018 17:12:30
Comments		
Input	Data	\\gweng.gmadtree.gmit.ie\STAFF\Benoit.Houeix\Documents\ST.sav
	Active Dataset	DataSet1
	Filter	<none>
	Weight	<none>
	Split File	<none>
	N of Rows in Working Data File	45
Missing Value Handling	Definition of Missing	User defined missing values are treated as missing.
	Cases Used	Statistics for each analysis are based on the cases with no missing or out-of-range data for any variable in the analysis.
Syntax		T-TEST GROUPS=Sample('Rno' 'ASF') /MISSING=ANALYSIS /VARIABLES=value1 /CRITERIA=CI(.95).
Resources	Processor Time	00:00:00.03
	Elapsed Time	00:00:00.03

Group Statistics

	Sample	N	Mean	Std. Deviation	Std. Error Mean
SNA-I	Rno	3	2.19693	.126660	.073127

ASF	3	.60230	.008643	.004990

Independent Samples Test

		Levene's Test for Equality of Variances		t-test for Equality of Means	
		F	Sig.	t	df
SNA-I	Equal variances assumed	4.760	.095	21.756	4
	Equal variances not assumed			21.756	2.019

Independent Samples Test

		t-test for Equality of Means			
					95% Confidence Interval of the Difference
		Sig. (2-tailed)	Mean Difference	Std. Error Difference	Lower
SNA-I	Equal variances assumed	.000	1.594633	.073297	1.391128
	Equal variances not assumed	.002	1.594633	.073297	1.282034

Independent Samples Test

		t-test for Equality of Means
		95% Confidence Interval of the Difference
		Upper
SNA-I	Equal variances assumed	1.798139
	Equal variances not assumed	1.907233

T-Test: mST6 (commercial control)

Notes

Output Created		11-JUL-2018 17:12:47
Comments		
Input	Data	\\gweng.gmadtree.gmit.ie\STAFF\Benoit.Houeix\Documents\ST.sav
	Active Dataset	DataSet1
	Filter	<none>
	Weight	<none>
	Split File	<none>

	N of Rows in Working Data File	45
Missing Value Handling	Definition of Missing	User defined missing values are treated as missing.
	Cases Used	Statistics for each analysis are based on the cases with no missing or out-of-range data for any variable in the analysis.
Syntax		T-TEST GROUPS=Sample('Ctrl' 'ASF')<br>/MISSING=ANALYSIS<br>/VARIABLES=value1<br>/CRITERIA=CI(.95).
Resources	Processor Time	00:00:00.00
	Elapsed Time	00:00:00.02

Group Statistics

	Sample	N	Mean	Std. Deviation	Std. Error Mean
SNA-I	Ctrl	3	2.64073	.153787	.088789
	ASF	3	.60230	.008643	.004990

Independent Samples Test

		Levene's Test for Equality of Variances		t-test for Equality of Means	
		F	Sig.	t	df
SNA-I	Equal variances assumed	6.047	.070	22.922	4
	Equal variances not assumed			22.922	2.013

Independent Samples Test

		t-test for Equality of Means			
					95% Confidence Interval of the Difference
		Sig. (2-tailed)	Mean Difference	Std. Error Difference	Lower
SNA-I	Equal variances assumed	.000	2.038433	.088929	1.791526
	Equal variances not assumed	.002	2.038433	.088929	1.658095

Independent Samples Test

		t-test for Equality of Means
		95% Confidence Interval of the Difference
		Upper
SNA-I	Equal variances assumed	2.285340
	Equal variances not assumed	2.418772

T-Test: hST3 A2

Notes

Output Created		11-JUL-2018 17:17:16
Comments		
Input	Data	\\gweng.gmadtree.gmit.ie\STAFF\Benoit.Houeix\Documents\ST.sav
	Active Dataset	DataSet1
	Filter	<none>
	Weight	<none>
	Split File	<none>
	N of Rows in Working Data File	45
Missing Value Handling	Definition of Missing	User defined missing values are treated as missing.
	Cases Used	Statistics for each analysis are based on the cases with no missing or out-of-range data for any variable in the analysis.
Syntax		T-TEST GROUPS=Sample('ST3A2' 'ASF') /MISSING=ANALYSIS /VARIABLES=value1 /CRITERIA=CI(.95).
Resources	Processor Time	00:00:00.00
	Elapsed Time	00:00:00.00

Group Statistics

	Sample	N	Mean	Std. Deviation	Std. Error Mean
SNA-I	ST3A2	3	.70623	.065623	.037887
	ASF	3	.60230	.008643	.004990

Independent Samples Test

		Levene's Test for Equality of Variances		t-test for Equality of Means	
		F	Sig.	t	df
SNA-I	Equal variances assumed	8.227	.046	2.720	4
	Equal variances not assumed			2.720	2.069

Independent Samples Test

		t-test for Equality of Means			
					95% Confidence Interval of the Difference
		Sig. (2-tailed)	Mean Difference	Std. Error Difference	Lower
SNA-I	Equal variances assumed	.053	.103933	.038215	-.002167
	Equal variances not assumed	.109	.103933	.038215	-.055320

Independent Samples Test

		t-test for Equality of Means
		95% Confidence Interval of the Difference
		Upper
SNA-I	Equal variances assumed	.210034
	Equal variances not assumed	.263187

T-Test: hST3 A1

Notes

Output Created		11-JUL-2018 17:19:54
Comments		
Input	Data	\\gweng.gmadtree.gmit.ie\STAFF\Benoit.Houeix\Documents\ST.sav
	Active Dataset	DataSet1
	Filter	<none>
	Weight	<none>
	Split File	<none>

	N of Rows in Working Data File	45
Missing Value Handling	Definition of Missing	User defined missing values are treated as missing.
	Cases Used	Statistics for each analysis are based on the cases with no missing or out-of-range data for any variable in the analysis.
Syntax		T-TEST GROUPS=Sample('ST3A1' 'ASF') /MISSING=ANALYSIS /VARIABLES=value1 /CRITERIA=CI(.95).
Resources	Processor Time	00:00:00.00
	Elapsed Time	00:00:00.02

Group Statistics

	Sample	N	Mean	Std. Deviation	Std. Error Mean
SNA-I	ST3A1	3	.80123	.033994	.019626
	ASF	3	.60230	.008643	.004990

Independent Samples Test

		Levene's Test for Equality of Variances		t-test for Equality of Means	
		F	Sig.	t	df
SNA-I	Equal variances assumed	7.959	.048	9.823	4
	Equal variances not assumed			9.823	2.258

Independent Samples Test

		t-test for Equality of Means			
		Sig. (2-tailed)	Mean Difference	Std. Error Difference	95% Confidence Interval of the Difference Lower
SNA-I	Equal variances assumed	.001	.198933	.020251	.142708
	Equal variances not assumed	.007	.198933	.020251	.120666

Independent Samples Test

		t-test for Equality of Means
		95% Confidence Interval of the Difference
		Upper
SNA-I	Equal variances assumed	.255159
	Equal variances not assumed	.277201

T-Test: zST3

Notes

Output Created		11-JUL-2018 17:20:13
Comments		
Input	Data	\\gweng.gmadtree.gmit.ie\STAFF\Benoit.Houeix\Documents\ST.sav
	Active Dataset	DataSet1
	Filter	<none>
	Weight	<none>
	Split File	<none>
	N of Rows in Working Data File	45
Missing Value Handling	Definition of Missing	User defined missing values are treated as missing.
	Cases Used	Statistics for each analysis are based on the cases with no missing or out-of-range data for any variable in the analysis.
Syntax		T-TEST GROUPS=Sample('ST3H6' 'ASF')<br>/MISSING=ANALYSIS<br>/VARIABLES=value1<br>/CRITERIA=CI(.95).
Resources	Processor Time	00:00:00.02
	Elapsed Time	00:00:00.03

Group Statistics

	Sample	N	Mean	Std. Deviation	Std. Error Mean
SNA-I	ST3H6	3	.83217	.074289	.042891
	ASF	3	.60230	.008643	.004990

Independent Samples Test

		Levene's Test for Equality of Variances		t-test for Equality of Means	
		F	Sig.	t	df
SNA-I	Equal variances assumed	5.627	.077	5.323	4
	Equal variances not assumed			5.323	2.054

Independent Samples Test

		t-test for Equality of Means			
					95% Confidence Interval of the Difference
		Sig. (2-tailed)	Mean Difference	Std. Error Difference	Lower
SNA-I	Equal variances assumed	.006	.229867	.043180	.109979
	Equal variances not assumed	.032	.229867	.043180	.048691

Independent Samples Test

		t-test for Equality of Means
		95% Confidence Interval of the Difference
		Upper
SNA-I	Equal variances assumed	.349755
	Equal variances not assumed	.411042

T-Test: hST6Gal2

Notes

Output Created	11-JUL-2018 17:20:50
Comments	

Input	Data	\\gweng.gmadtree.gmit.ie\STAFF\Benoit.Houeix\Documents\ST.sav
	Active Dataset	DataSet1
	Filter	<none>
	Weight	<none>
	Split File	<none>
	N of Rows in Working Data File	45
Missing Value Handling	Definition of Missing	User defined missing values are treated as missing.
	Cases Used	Statistics for each analysis are based on the cases with no missing or out-of-range data for any variable in the analysis.
Syntax		T-TEST GROUPS=Sample('ST2' 'ASF') /MISSING=ANALYSIS /VARIABLES=value1 /CRITERIA=CI(.95).
Resources	Processor Time	00:00:00.00
	Elapsed Time	00:00:00.03

Group Statistics

	Sample	N	Mean	Std. Deviation	Std. Error Mean
SNA-I	ST2	3	.58790	.053927	.031135
	ASF	3	.60230	.008643	.004990

Independent Samples Test

		Levene's Test for Equality of Variances		t-test for Equality of Means	
		F	Sig.	t	df
SNA-I	Equal variances assumed	7.825	.049	-.457	4
	Equal variances not assumed			-.457	2.103

Independent Samples Test

	t-test for Equality of Means

		Sig. (2-tailed)	Mean Difference	Std. Error Difference	95% Confidence Interval of the Difference
					Lower
SNA-I	Equal variances assumed	.672	-.014400	.031532	-.101948
	Equal variances not assumed	.691	-.014400	.031532	-.143917

Independent Samples Test

		t-test for Equality of Means
		95% Confidence Interval of the Difference
		Upper
SNA-I	Equal variances assumed	.073148
	Equal variances not assumed	.115117

Notes

Output Created		11-JUL-2018 17:24:09
Comments		
Input	Data	\\gweng.gmadtree.gmit.ie\STAFF\Benoit.Houeix\Documents\ST.sav
	Active Dataset	DataSet1
	Filter	<none>
	Weight	<none>
	Split File	<none>
	N of Rows in Working Data File	45
Syntax		NPTESTS /INDEPENDENT TEST (value1 value2) GROUP (Sample) KRUSKAL_WALLIS(COMPARE=PAIRWISE) /MISSING SCOPE=ANALYSIS USERMISSING=EXCLUDE /CRITERIA ALPHA=0.05 CILEVEL=95.
Resources	Processor Time	00:00:00.11
	Elapsed Time	00:00:00.13
