# Supplementary material for: Engineering of CHO cells for the production of vertebrate recombinant sialyltransferases
Source: PeerJ. 2019 Feb 11;7:e5788. doi: 10.7717/peerj.5788 (PMC6375257; doi:10.7717/peerj.5788)
Supplement: Supplemental Information 14 — ASF, reacted with each of the sialyltransferase preparations (37 ° C, 16 h), was bound to biotinylated SNA-I lectin. Binding to Neu5Ac on the ASF was detected with HRP-streptavidin. Expt 1 (black bars–Figure 6A) included ST6Gal I, ST3Gal IV and ST6Gal II preparations. Expt 2 (grey bars–Figure 6A) included only ST6Gal I preparations. Each sample was analysed for binding with biotinylated RCA I lectin (Figure 6B). All values are relative to ASF. Each sample was assayed in triplicate. Statistics–part B (RCA-I ANOVA). [file peerj-07-5788-s014.pdf]

```
UNIANOVA RCA_I BY Sample
  /METHOD=SSTYPE(3)
  /INTERCEPT=INCLUDE
  /POSTHOC=Sample(BONFERRONI)
  /PLOT=PROFILE(Sample)
  /EMMEANS=TABLES(Sample)
  /PRINT=HOMOGENEITY DESCRIPTIVE
  /CRITERIA=ALPHA(.05)
  /DESIGN=Sample.
```

Univariate Analysis of Variance

Label for Statistics	Corresponding in Manuscript
ASF	ASF (Asialofetuin)
FET	FET (Fetuin)
A5	hST6 A5
B1	hST6 B1
G1	hST6
Dre	zST6
Gac	sST6
Tru	fST6
Gga	cST6
Rno	rST6
Ctrl	mST6 (commercial mouse enzyme)
ST3A2	hST3 A2
ST3A1	hST3 A2
ST3H6	zST3
ST2	hST6Gal2

Notes

Output Created		12-JUL-2018 14:55:49
Comments		
Input	Data	\\gweng.gmadtree.gmit.ie\STAFF\Benoit.Houeix\Documents\ST.sav
	Active Dataset	DataSet1
	Filter	<none>
	Weight	<none>
	Split File	<none>
	N of Rows in Working Data File	48

Missing Value Handling	Definition of Missing	User-defined missing values are treated as missing.
	Cases Used	Statistics are based on all cases with valid data for all variables in the model.
Syntax		UNIANOVA RCA_I BY Sample<br>/METHOD=SSTYPE(3)<br>/INTERCEPT=INCLUDE<br>/POSTHOC=Sample(BONFERRONI)<br>/PLOT=PROFILE(Sample)<br>/EMMEANS=TABLES(Sample)<br>/PRINT=HOMOGENEITY DESCRIPTIVE<br>/CRITERIA=ALPHA(.05)<br>/DESIGN=Sample.
Resources	Processor Time	00:00:00.17
	Elapsed Time	00:00:00.20

Between-Subjects Factors

		Value Label	N
Sample	A5	A5	3
	ASF	ASF	3
	B1	B1	3
	Ctrl	Ctrl	3
	Dre	Dre	3
	G1	G1	3
	Gac	Gac	3
	Gga	Gga	3
	Rno	Rno	3
	Tru	Tru	3

Descriptive Statistics

Dependent Variable: RCA_I

Sample	Mean	Std. Deviation	N
A5	-.6163	.09645	3
ASF	-.0001	.11230	3
B1	-.6739	.08488	3
Ctrl	-.7016	.04817	3
Dre	-.4966	.14420	3

G1	-.5872	.18771	3
Gac	-.4787	.09506	3
Gga	-.7201	.10144	3
Rno	-.5594	.08450	3
Tru	-.4295	.04832	3
Total	-.5263	.22048	30

Levene's Test of Equality of Error Variances[a]

Dependent Variable: RCA_I

F	df1	df2	Sig.
1.589	9	20	.186

Tests the null hypothesis that the error variance of the dependent variable is equal across groups.[a]

a. Design: Intercept + Sample

Tests of Between-Subjects Effects

Dependent Variable: RCA_I

Source	Type III Sum of Squares	df	Mean Square	F	Sig.
Corrected Model	1.177[a]	9	.131	11.249	.000
Intercept	8.311	1	8.311	714.805	.000
Sample	1.177	9	.131	11.249	.000
Error	.233	20	.012		
Total	9.721	30			
Corrected Total	1.410	29			

a. R Squared = .835 (Adjusted R Squared = .761)

Estimated Marginal Means

Sample

Dependent Variable: RCA_I

Sample	Mean	Std. Error	95% Confidence Interval	
			Lower Bound	Upper Bound

A5	-.616	.062	-.746	-.486
ASF	-1.000E-4	.062	-.130	.130
B1	-.674	.062	-.804	-.544
Ctrl	-.702	.062	-.831	-.572
Dre	-.497	.062	-.626	-.367
G1	-.587	.062	-.717	-.457
Gac	-.479	.062	-.609	-.349
Gga	-.720	.062	-.850	-.590
Rno	-.559	.062	-.689	-.430
Tru	-.429	.062	-.559	-.300

Post Hoc Tests

Sample

Multiple Comparisons

Dependent Variable: RCA_I

Bonferroni

(I) Sample	(J) Sample	Mean Difference (I-J)	Std. Error	Sig.	95% Confidence Interval	
					Lower Bound	Upper Bound
A5	ASF	-.6162*	.08804	.000	-.9511	-.2813
	B1	.0576	.08804	1.000	-.2774	.3925
	Ctrl	.0853	.08804	1.000	-.2496	.4203
	Dre	-.1197	.08804	1.000	-.4547	.2152
	G1	-.0291	.08804	1.000	-.3640	.3058
	Gac	-.1376	.08804	1.000	-.4725	.1974
	Gga	.1038	.08804	1.000	-.2311	.4387
	Rno	-.0569	.08804	1.000	-.3918	.2781
	Tru	-.1868	.08804	1.000	-.5218	.1481
ASF	A5	.6162*	.08804	.000	.2813	.9511
	B1	.6738*	.08804	.000	.3388	1.0087
	Ctrl	.7015*	.08804	.000	.3666	1.0365
	Dre	.4965*	.08804	.001	.1615	.8314

	G1	.5871*	.08804	.000	.2522	.9220
	Gac	.4786*	.08804	.001	.1437	.8136
	Gga	.7200*	.08804	.000	.3851	1.0549
	Rno	.5593*	.08804	.000	.2244	.8943
	Tru	.4294*	.08804	.004	.0944	.7643
B1	A5	-.0576	.08804	1.000	-.3925	.2774
	ASF	-.6738*	.08804	.000	-1.0087	-.3388
	Ctrl	.0278	.08804	1.000	-.3072	.3627
	Dre	-.1773	.08804	1.000	-.5122	.1576
	G1	-.0867	.08804	1.000	-.4216	.2483
	Gac	-.1951	.08804	1.000	-.5301	.1398
	Gga	.0462	.08804	1.000	-.2887	.3812
	Rno	-.1144	.08804	1.000	-.4494	.2205
	Tru	-.2444	.08804	.525	-.5793	.0905
Ctrl	A5	-.0853	.08804	1.000	-.4203	.2496
	ASF	-.7015*	.08804	.000	-1.0365	-.3666
	B1	-.0278	.08804	1.000	-.3627	.3072
	Dre	-.2051	.08804	1.000	-.5400	.1299
	G1	-.1144	.08804	1.000	-.4494	.2205
	Gac	-.2229	.08804	.893	-.5578	.1120
	Gga	.0185	.08804	1.000	-.3165	.3534
	Rno	-.1422	.08804	1.000	-.4771	.1927
	Tru	-.2722	.08804	.259	-.6071	.0628
Dre	A5	.1197	.08804	1.000	-.2152	.4547
	ASF	-.4965*	.08804	.001	-.8314	-.1615
	B1	.1773	.08804	1.000	-.1576	.5122
	Ctrl	.2051	.08804	1.000	-.1299	.5400
	G1	.0906	.08804	1.000	-.2443	.4256
	Gac	-.0178	.08804	1.000	-.3528	.3171
	Gga	.2235	.08804	.879	-.1114	.5585
	Rno	.0629	.08804	1.000	-.2721	.3978
	Tru	-.0671	.08804	1.000	-.4020	.2678
G1	A5	.0291	.08804	1.000	-.3058	.3640
	ASF	-.5871*	.08804	.000	-.9220	-.2522
	B1	.0867	.08804	1.000	-.2483	.4216
	Ctrl	.1144	.08804	1.000	-.2205	.4494
	Dre	-.0906	.08804	1.000	-.4256	.2443
	Gac	-.1085	.08804	1.000	-.4434	.2265
	Gga	.1329	.08804	1.000	-.2020	.4678
	Rno	-.0278	.08804	1.000	-.3627	.3072
	Tru	-.1577	.08804	1.000	-.4927	.1772

Gac	A5	.1376	.08804	1.000	-.1974	.4725
	ASF	-.4786*	.08804	.001	-.8136	-.1437
	B1	.1951	.08804	1.000	-.1398	.5301
	Ctrl	.2229	.08804	.893	-.1120	.5578
	Dre	.0178	.08804	1.000	-.3171	.3528
	G1	.1085	.08804	1.000	-.2265	.4434
	Gga	.2414	.08804	.566	-.0936	.5763
	Rno	.0807	.08804	1.000	-.2542	.4156
	Tru	-.0493	.08804	1.000	-.3842	.2857
Gga	A5	-.1038	.08804	1.000	-.4387	.2311
	ASF	-.7200*	.08804	.000	-1.0549	-.3851
	B1	-.0462	.08804	1.000	-.3812	.2887
	Ctrl	-.0185	.08804	1.000	-.3534	.3165
	Dre	-.2235	.08804	.879	-.5585	.1114
	G1	-.1329	.08804	1.000	-.4678	.2020
	Gac	-.2414	.08804	.566	-.5763	.0936
	Rno	-.1607	.08804	1.000	-.4956	.1743
	Tru	-.2906	.08804	.161	-.6256	.0443
Rno	A5	.0569	.08804	1.000	-.2781	.3918
	ASF	-.5593*	.08804	.000	-.8943	-.2244
	B1	.1144	.08804	1.000	-.2205	.4494
	Ctrl	.1422	.08804	1.000	-.1927	.4771
	Dre	-.0629	.08804	1.000	-.3978	.2721
	G1	.0278	.08804	1.000	-.3072	.3627
	Gac	-.0807	.08804	1.000	-.4156	.2542
	Gga	.1607	.08804	1.000	-.1743	.4956
	Tru	-.1300	.08804	1.000	-.4649	.2050
Tru	A5	.1868	.08804	1.000	-.1481	.5218
	ASF	-.4294*	.08804	.004	-.7643	-.0944
	B1	.2444	.08804	.525	-.0905	.5793
	Ctrl	.2722	.08804	.259	-.0628	.6071
	Dre	.0671	.08804	1.000	-.2678	.4020
	G1	.1577	.08804	1.000	-.1772	.4927
	Gac	.0493	.08804	1.000	-.2857	.3842
	Gga	.2906	.08804	.161	-.0443	.6256
	Rno	.1300	.08804	1.000	-.2050	.4649

Based on observed means.

The error term is Mean Square(Error) = .012.

*. The mean difference is significant at the .05 level.

Profile Plots

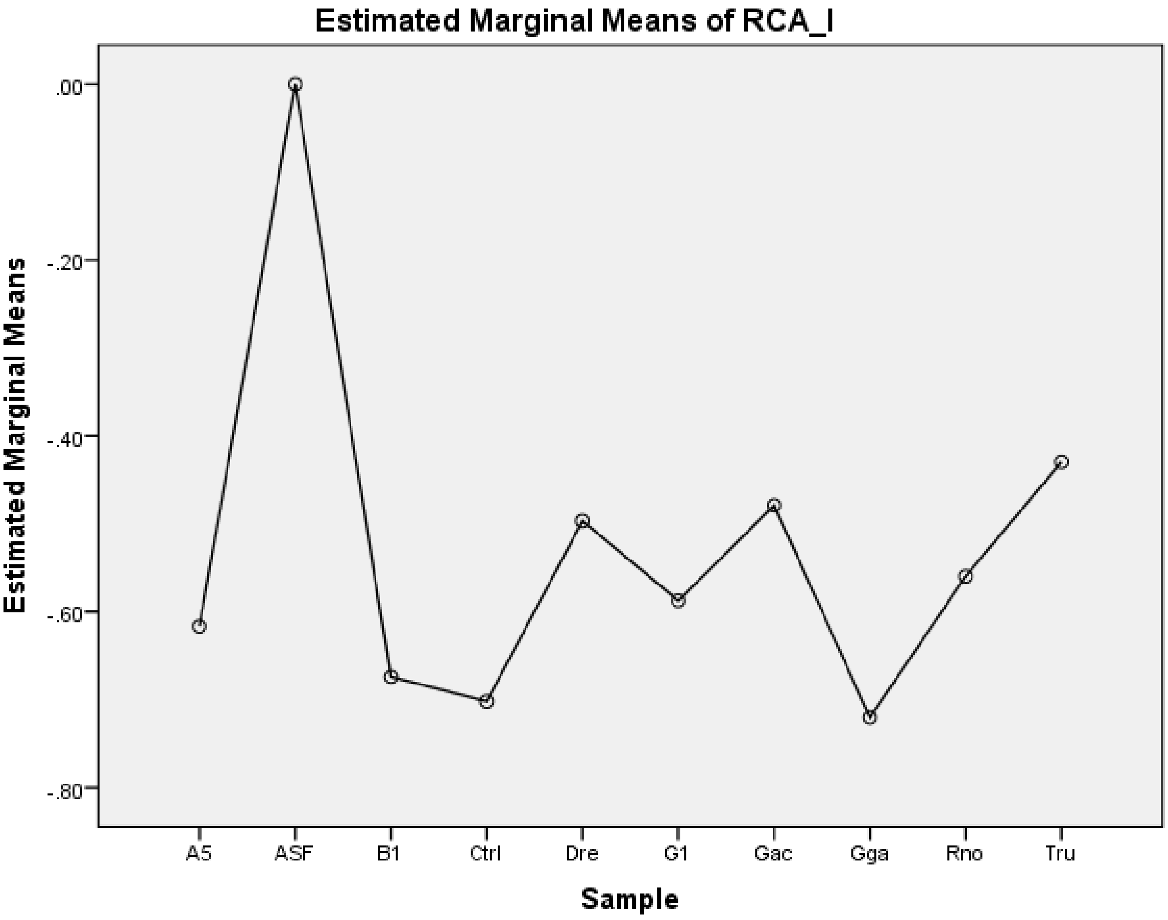


```
UNIANOVA value2 BY Sample
  /METHOD=SSTYPE(3)
  /INTERCEPT=INCLUDE
  /POSTHOC=Sample(BONFERRONI)
  /PLOT=PROFILE(Sample)
  /EMMEANS=TABLES(Sample)
  /PRINT=HOMOGENEITY DESCRIPTIVE
  /CRITERIA=ALPHA(.05)
  /DESIGN=Sample.
```

Univariate Analysis of Variance - RCA-I values

Notes

Output Created		12-JUL-2018 14:57:35
Comments		
Input	Data	\\gweng.gmadtree.gmit.ie\STAFF\Benoit.Houeix\Documents\ST.sav
	Active Dataset	DataSet1
	Filter	<none>
	Weight	<none>
	Split File	<none>
	N of Rows in Working Data File	48
Missing Value Handling	Definition of Missing	User-defined missing values are treated as missing.
	Cases Used	Statistics are based on all cases with valid data for all variables in the model.
Syntax		UNIANOVA value2 BY Sample<br>/METHOD=SSTYPE(3)<br>/INTERCEPT=INCLUDE<br>/POSTHOC=Sample(BONFERRONI)<br>/PLOT=PROFILE(Sample)<br>/EMMEANS=TABLES(Sample)<br>/PRINT=HOMOGENEITY DESCRIPTIVE<br>/CRITERIA=ALPHA(.05)<br>/DESIGN=Sample.
Resources	Processor Time	00:00:00.19
	Elapsed Time	00:00:00.28

Between-Subjects Factors

		Value Label	N
Sample	A5	A5	3
	ASF	ASF	3
	B1	B1	3
	Ctrl	Ctrl	3
	Dre	Dre	3
	FET	FET	3
	G1	G1	3

	Gac	Gac	3
	Gga	Gga	3
	Rno	Rno	3
	ST2	ST2	3
	ST3A1	ST3A1	3
	ST3A2	ST3A2	3
	ST3H6	ST3H6	3
	Tru	Tru	3

Descriptive Statistics

Dependent Variable: RCA-I

Sample	Mean	Std. Deviation	N
A5	2.72470	.096450	3
ASF	3.34090	.112297	3
B1	2.66713	.084883	3
Ctrl	2.63937	.048172	3
Dre	2.84443	.144199	3
FET	2.97617	.064672	3
G1	2.75380	.187707	3
Gac	2.86227	.095064	3
Gga	2.62090	.101442	3
Rno	2.78157	.084502	3
ST2	3.29277	.083380	3
ST3A1	2.74630	.159049	3
ST3A2	2.88480	.041250	3
ST3H6	2.83923	.263972	3
Tru	2.91153	.048318	3
Total	2.85906	.230180	45

Levene's Test of Equality of Error Variances[a]

Dependent Variable: RCA-I

F	df1	df2	Sig.
2.669	14	30	.012

Tests the null hypothesis that the error variance of the dependent variable is equal across groups.[a]

a. Design: Intercept + Sample

Tests of Between-Subjects Effects

Dependent Variable: RCA-I

Source	Type III Sum of Squares	df	Mean Square	F	Sig.
Corrected Model	1.883[a]	14	.135	9.004	.000
Intercept	367.840	1	367.840	24622.900	.000
Sample	1.883	14	.135	9.004	.000
Error	.448	30	.015		
Total	370.171	45			
Corrected Total	2.331	44			

a. R Squared = .808 (Adjusted R Squared = .718)

Estimated Marginal Means

Sample

Dependent Variable: RCA-I

Sample	Mean	Std. Error	95% Confidence Interval	
			Lower Bound	Upper Bound
A5	2.725	.071	2.581	2.869
ASF	3.341	.071	3.197	3.485
B1	2.667	.071	2.523	2.811
Ctrl	2.639	.071	2.495	2.783
Dre	2.844	.071	2.700	2.989
FET	2.976	.071	2.832	3.120
G1	2.754	.071	2.610	2.898
Gac	2.862	.071	2.718	3.006
Gga	2.621	.071	2.477	2.765
Rno	2.782	.071	2.637	2.926
ST2	3.293	.071	3.149	3.437
ST3A1	2.746	.071	2.602	2.890
ST3A2	2.885	.071	2.741	3.029
ST3H6	2.839	.071	2.695	2.983
Tru	2.912	.071	2.767	3.056

Post Hoc Tests

Sample

Multiple Comparisons

Dependent Variable: RCA-I

Bonferroni

(I) Sample	(J) Sample	Mean Difference (I-J)	Std. Error	Sig.	95% Confidence Interval Lower Bound	Upper Bound
A5	ASF	-.61620*	.099796	.000	-1.00735	-.22505
	B1	.05757	.099796	1.000	-.33358	.44872
	Ctrl	.08533	.099796	1.000	-.30582	.47648
	Dre	-.11973	.099796	1.000	-.51088	.27142
	FET	-.25147	.099796	1.000	-.64262	.13968
	G1	-.02910	.099796	1.000	-.42025	.36205
	Gac	-.13757	.099796	1.000	-.52872	.25358
	Gga	.10380	.099796	1.000	-.28735	.49495
	Rno	-.05687	.099796	1.000	-.44802	.33428
	ST2	-.56807*	.099796	.000	-.95922	-.17692
	ST3A1	-.02160	.099796	1.000	-.41275	.36955
	ST3A2	-.16010	.099796	1.000	-.55125	.23105
	ST3H6	-.11453	.099796	1.000	-.50568	.27662
	Tru	-.18683	.099796	1.000	-.57798	.20432
ASF	A5	.61620*	.099796	.000	.22505	1.00735
	B1	.67377*	.099796	.000	.28262	1.06492
	Ctrl	.70153*	.099796	.000	.31038	1.09268
	Dre	.49647*	.099796	.003	.10532	.88762
	FET	.36473	.099796	.103	-.02642	.75588
	G1	.58710*	.099796	.000	.19595	.97825
	Gac	.47863*	.099796	.004	.08748	.86978
	Gga	.72000*	.099796	.000	.32885	1.11115
	Rno	.55933*	.099796	.000	.16818	.95048
	ST2	.04813	.099796	1.000	-.34302	.43928
	ST3A1	.59460*	.099796	.000	.20345	.98575

	ST3A2	.45610*	.099796	.008	.06495	.84725
	ST3H6	.50167*	.099796	.002	.11052	.89282
	Tru	.42937*	.099796	.017	.03822	.82052
B1	A5	-.05757	.099796	1.000	-.44872	.33358
	ASF	-.67377*	.099796	.000	-1.06492	-.28262
	Ctrl	.02777	.099796	1.000	-.36338	.41892
	Dre	-.17730	.099796	1.000	-.56845	.21385
	FET	-.30903	.099796	.443	-.70018	.08212
	G1	-.08667	.099796	1.000	-.47782	.30448
	Gac	-.19513	.099796	1.000	-.58628	.19602
	Gga	.04623	.099796	1.000	-.34492	.43738
	Rno	-.11443	.099796	1.000	-.50558	.27672
	ST2	-.62563*	.099796	.000	-1.01678	-.23448
	ST3A1	-.07917	.099796	1.000	-.47032	.31198
	ST3A2	-.21767	.099796	1.000	-.60882	.17348
	ST3H6	-.17210	.099796	1.000	-.56325	.21905
	Tru	-.24440	.099796	1.000	-.63555	.14675
Ctrl	A5	-.08533	.099796	1.000	-.47648	.30582
	ASF	-.70153*	.099796	.000	-1.09268	-.31038
	B1	-.02777	.099796	1.000	-.41892	.36338
	Dre	-.20507	.099796	1.000	-.59622	.18608
	FET	-.33680	.099796	.216	-.72795	.05435
	G1	-.11443	.099796	1.000	-.50558	.27672
	Gac	-.22290	.099796	1.000	-.61405	.16825
	Gga	.01847	.099796	1.000	-.37268	.40962
	Rno	-.14220	.099796	1.000	-.53335	.24895
	ST2	-.65340*	.099796	.000	-1.04455	-.26225
	ST3A1	-.10693	.099796	1.000	-.49808	.28422
	ST3A2	-.24543	.099796	1.000	-.63658	.14572
	ST3H6	-.19987	.099796	1.000	-.59102	.19128
	Tru	-.27217	.099796	1.000	-.66332	.11898
Dre	A5	.11973	.099796	1.000	-.27142	.51088
	ASF	-.49647*	.099796	.003	-.88762	-.10532
	B1	.17730	.099796	1.000	-.21385	.56845
	Ctrl	.20507	.099796	1.000	-.18608	.59622
	FET	-.13173	.099796	1.000	-.52288	.25942
	G1	.09063	.099796	1.000	-.30052	.48178
	Gac	-.01783	.099796	1.000	-.40898	.37332
	Gga	.22353	.099796	1.000	-.16762	.61468
	Rno	.06287	.099796	1.000	-.32828	.45402
	ST2	-.44833*	.099796	.010	-.83948	-.05718

	ST3A1	.09813	.099796	1.000	-.29302	.48928
	ST3A2	-.04037	.099796	1.000	-.43152	.35078
	ST3H6	.00520	.099796	1.000	-.38595	.39635
	Tru	-.06710	.099796	1.000	-.45825	.32405
FET	A5	.25147	.099796	1.000	-.13968	.64262
	ASF	-.36473	.099796	.103	-.75588	.02642
	B1	.30903	.099796	.443	-.08212	.70018
	Ctrl	.33680	.099796	.216	-.05435	.72795
	Dre	.13173	.099796	1.000	-.25942	.52288
	G1	.22237	.099796	1.000	-.16878	.61352
	Gac	.11390	.099796	1.000	-.27725	.50505
	Gga	.35527	.099796	.132	-.03588	.74642
	Rno	.19460	.099796	1.000	-.19655	.58575
	ST2	-.31660	.099796	.365	-.70775	.07455
	ST3A1	.22987	.099796	1.000	-.16128	.62102
	ST3A2	.09137	.099796	1.000	-.29978	.48252
	ST3H6	.13693	.099796	1.000	-.25422	.52808
	Tru	.06463	.099796	1.000	-.32652	.45578
G1	A5	.02910	.099796	1.000	-.36205	.42025
	ASF	-.58710*	.099796	.000	-.97825	-.19595
	B1	.08667	.099796	1.000	-.30448	.47782
	Ctrl	.11443	.099796	1.000	-.27672	.50558
	Dre	-.09063	.099796	1.000	-.48178	.30052
	FET	-.22237	.099796	1.000	-.61352	.16878
	Gac	-.10847	.099796	1.000	-.49962	.28268
	Gga	.13290	.099796	1.000	-.25825	.52405
	Rno	-.02777	.099796	1.000	-.41892	.36338
	ST2	-.53897*	.099796	.001	-.93012	-.14782
	ST3A1	.00750	.099796	1.000	-.38365	.39865
	ST3A2	-.13100	.099796	1.000	-.52215	.26015
	ST3H6	-.08543	.099796	1.000	-.47658	.30572
	Tru	-.15773	.099796	1.000	-.54888	.23342
Gac	A5	.13757	.099796	1.000	-.25358	.52872
	ASF	-.47863*	.099796	.004	-.86978	-.08748
	B1	.19513	.099796	1.000	-.19602	.58628
	Ctrl	.22290	.099796	1.000	-.16825	.61405
	Dre	.01783	.099796	1.000	-.37332	.40898
	FET	-.11390	.099796	1.000	-.50505	.27725
	G1	.10847	.099796	1.000	-.28268	.49962
	Gga	.24137	.099796	1.000	-.14978	.63252
	Rno	.08070	.099796	1.000	-.31045	.47185

	ST2	-.43050*	.099796	.017	-.82165	-.03935
	ST3A1	.11597	.099796	1.000	-.27518	.50712
	ST3A2	-.02253	.099796	1.000	-.41368	.36862
	ST3H6	.02303	.099796	1.000	-.36812	.41418
	Tru	-.04927	.099796	1.000	-.44042	.34188
Gga	A5	-.10380	.099796	1.000	-.49495	.28735
	ASF	-.72000*	.099796	.000	-1.11115	-.32885
	B1	-.04623	.099796	1.000	-.43738	.34492
	Ctrl	-.01847	.099796	1.000	-.40962	.37268
	Dre	-.22353	.099796	1.000	-.61468	.16762
	FET	-.35527	.099796	.132	-.74642	.03588
	G1	-.13290	.099796	1.000	-.52405	.25825
	Gac	-.24137	.099796	1.000	-.63252	.14978
	Rno	-.16067	.099796	1.000	-.55182	.23048
	ST2	-.67187*	.099796	.000	-1.06302	-.28072
	ST3A1	-.12540	.099796	1.000	-.51655	.26575
	ST3A2	-.26390	.099796	1.000	-.65505	.12725
	ST3H6	-.21833	.099796	1.000	-.60948	.17282
	Tru	-.29063	.099796	.705	-.68178	.10052
Rno	A5	.05687	.099796	1.000	-.33428	.44802
	ASF	-.55933*	.099796	.000	-.95048	-.16818
	B1	.11443	.099796	1.000	-.27672	.50558
	Ctrl	.14220	.099796	1.000	-.24895	.53335
	Dre	-.06287	.099796	1.000	-.45402	.32828
	FET	-.19460	.099796	1.000	-.58575	.19655
	G1	.02777	.099796	1.000	-.36338	.41892
	Gac	-.08070	.099796	1.000	-.47185	.31045
	Gga	.16067	.099796	1.000	-.23048	.55182
	ST2	-.51120*	.099796	.002	-.90235	-.12005
	ST3A1	.03527	.099796	1.000	-.35588	.42642
	ST3A2	-.10323	.099796	1.000	-.49438	.28792
	ST3H6	-.05767	.099796	1.000	-.44882	.33348
	Tru	-.12997	.099796	1.000	-.52112	.26118
ST2	A5	.56807*	.099796	.000	.17692	.95922
	ASF	-.04813	.099796	1.000	-.43928	.34302
	B1	.62563*	.099796	.000	.23448	1.01678
	Ctrl	.65340*	.099796	.000	.26225	1.04455
	Dre	.44833*	.099796	.010	.05718	.83948
	FET	.31660	.099796	.365	-.07455	.70775
	G1	.53897*	.099796	.001	.14782	.93012
	Gac	.43050*	.099796	.017	.03935	.82165

	Gga	.67187*	.099796	.000	.28072	1.06302
	Rno	.51120*	.099796	.002	.12005	.90235
	ST3A1	.54647*	.099796	.001	.15532	.93762
	ST3A2	.40797*	.099796	.031	.01682	.79912
	ST3H6	.45353*	.099796	.009	.06238	.84468
	Tru	.38123	.099796	.066	-.00992	.77238
ST3A1	A5	.02160	.099796	1.000	-.36955	.41275
	ASF	-.59460*	.099796	.000	-.98575	-.20345
	B1	.07917	.099796	1.000	-.31198	.47032
	Ctrl	.10693	.099796	1.000	-.28422	.49808
	Dre	-.09813	.099796	1.000	-.48928	.29302
	FET	-.22987	.099796	1.000	-.62102	.16128
	G1	-.00750	.099796	1.000	-.39865	.38365
	Gac	-.11597	.099796	1.000	-.50712	.27518
	Gga	.12540	.099796	1.000	-.26575	.51655
	Rno	-.03527	.099796	1.000	-.42642	.35588
	ST2	-.54647*	.099796	.001	-.93762	-.15532
	ST3A2	-.13850	.099796	1.000	-.52965	.25265
	ST3H6	-.09293	.099796	1.000	-.48408	.29822
	Tru	-.16523	.099796	1.000	-.55638	.22592
ST3A2	A5	.16010	.099796	1.000	-.23105	.55125
	ASF	-.45610*	.099796	.008	-.84725	-.06495
	B1	.21767	.099796	1.000	-.17348	.60882
	Ctrl	.24543	.099796	1.000	-.14572	.63658
	Dre	.04037	.099796	1.000	-.35078	.43152
	FET	-.09137	.099796	1.000	-.48252	.29978
	G1	.13100	.099796	1.000	-.26015	.52215
	Gac	.02253	.099796	1.000	-.36862	.41368
	Gga	.26390	.099796	1.000	-.12725	.65505
	Rno	.10323	.099796	1.000	-.28792	.49438
	ST2	-.40797*	.099796	.031	-.79912	-.01682
	ST3A1	.13850	.099796	1.000	-.25265	.52965
	ST3H6	.04557	.099796	1.000	-.34558	.43672
	Tru	-.02673	.099796	1.000	-.41788	.36442
ST3H6	A5	.11453	.099796	1.000	-.27662	.50568
	ASF	-.50167*	.099796	.002	-.89282	-.11052
	B1	.17210	.099796	1.000	-.21905	.56325
	Ctrl	.19987	.099796	1.000	-.19128	.59102
	Dre	-.00520	.099796	1.000	-.39635	.38595
	FET	-.13693	.099796	1.000	-.52808	.25422
	G1	.08543	.099796	1.000	-.30572	.47658

	Gac	-.02303	.099796	1.000	-.41418	.36812
	Gga	.21833	.099796	1.000	-.17282	.60948
	Rno	.05767	.099796	1.000	-.33348	.44882
	ST2	-.45353*	.099796	.009	-.84468	-.06238
	ST3A1	.09293	.099796	1.000	-.29822	.48408
	ST3A2	-.04557	.099796	1.000	-.43672	.34558
	Tru	-.07230	.099796	1.000	-.46345	.31885
Tru	A5	.18683	.099796	1.000	-.20432	.57798
	ASF	-.42937*	.099796	.017	-.82052	-.03822
	B1	.24440	.099796	1.000	-.14675	.63555
	Ctrl	.27217	.099796	1.000	-.11898	.66332
	Dre	.06710	.099796	1.000	-.32405	.45825
	FET	-.06463	.099796	1.000	-.45578	.32652
	G1	.15773	.099796	1.000	-.23342	.54888
	Gac	.04927	.099796	1.000	-.34188	.44042
	Gga	.29063	.099796	.705	-.10052	.68178
	Rno	.12997	.099796	1.000	-.26118	.52112
	ST2	-.38123	.099796	.066	-.77238	.00992
	ST3A1	.16523	.099796	1.000	-.22592	.55638
	ST3A2	.02673	.099796	1.000	-.36442	.41788
	ST3H6	.07230	.099796	1.000	-.31885	.46345

Based on observed means.

The error term is Mean Square(Error) = .015.

*. The mean difference is significant at the .05 level.

Profile Plots

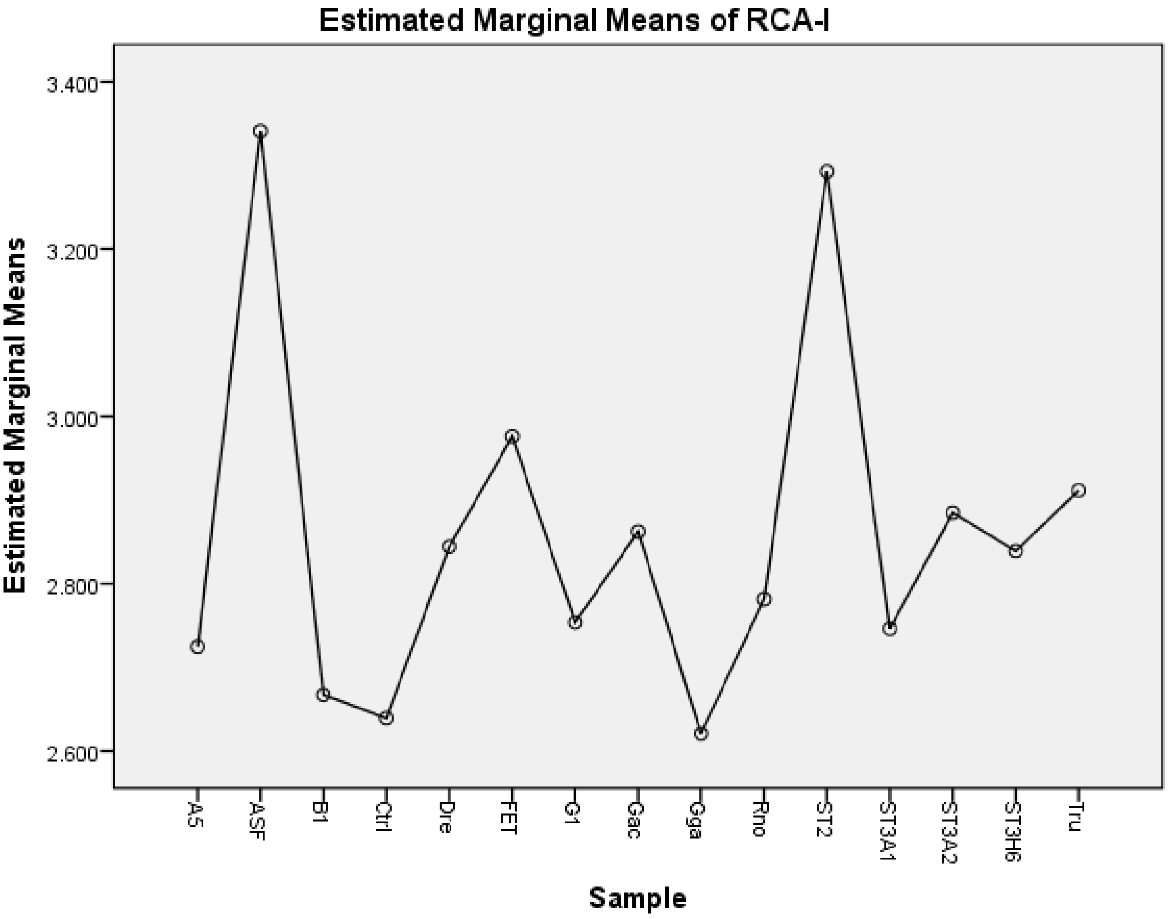
Estimated Marginal Means of RCA-I
Estimated Marginal Means
3.400
3.200
3.000
2.800
2.600
A5
ASF
B1
Ctrl
Dre
FET
G1
Gac
Gga
Rho
ST2
ST3A1
ST3A2
ST3H6
Tru
Sample
