# Supplementary material for: Engineering of CHO cells for the production of vertebrate recombinant sialyltransferases
Source: PeerJ. 2019 Feb 11;7:e5788. doi: 10.7717/peerj.5788 (PMC6375257; doi:10.7717/peerj.5788)
Supplement: Supplemental Information 15 — ASF, reacted with each of the sialyltransferase preparations (37 ° C, 16 h), was bound to biotinylated SNA-I lectin. Binding to Neu5Ac on the ASF was detected with HRP-streptavidin. Expt 1 (black bars–Figure 6A) included ST6Gal I, ST3Gal IV and ST6Gal II preparations. Expt 2 (grey bars–Figure 6A) included only ST6Gal I preparations. Each sample was analysed for binding with biotinylated RCA I lectin (Figure 6B). All values are relative to ASF. Each sample was assayed in triplicate. Statistics–part C (Post-hoc tests). [file peerj-07-5788-s015.pdf]

Statistics ELLA experiment – Figure 6

Levene's Test of Equality of Error Variances[a]

Dependent Variable: value1

F	df1	df2	Sig.
2.527	14	30	.016

Tests the null hypothesis that the error variance of the dependent variable is equal across groups.

a. Design: Intercept + Sample

Data not normal => **Non parametric tests**

Hypothesis Test Summary

	Null Hypothesis	Test	Sig.	Decision
1	The distribution of SNA-I is the same across categories of Sample.	Independent-Samples Kruskal-Wallis Test	.000	Reject the null hypothesis.
2	The distribution of RCA-I is the same across categories of Sample.	Independent-Samples Kruskal-Wallis Test	.006	Reject the null hypothesis.

Asymptotic significances are displayed. The significance level is .05.

Multivariate Tests[a]

Effect		Value	F	Hypothesis df	Error df	Sig.
Intercept	Pillai's Trace	.999	15233.281[b]	2.000	29.000	.000
	Wilks' Lambda	.001	15233.281[b]	2.000	29.000	.000
	Hotelling's Trace	1050.571	15233.281[b]	2.000	29.000	.000
	Roy's Largest Root	1050.571	15233.281[b]	2.000	29.000	.000
Sample	Pillai's Trace	1.768	16.326	28.000	60.000	.000
	Wilks' Lambda	.002	42.100[b]	28.000	58.000	.000
	Hotelling's Trace	103.516	103.516	28.000	56.000	.000
	Roy's Largest Root	100.015	214.317[c]	14.000	30.000	.000

a. Design: Intercept + Sample

b. Exact statistic

c. The statistic is an upper bound on F that yields a lower bound on the significance level.

Conclusion: Significant differences between samples

Figure 6: Legend

Label for Statistics	Corresponding in Manuscript
ASF	ASF (Asialofetuin)
FET	FET (Fetuin)
A5	hST6 A5
B1	hST6 B1
G1	hST6
Dre	zST6
Gac	sST6
Tru	fST6
Gga	cST6
Rno	rST6
Ctrl	mST6 (commercial mouse enzyme)
ST3A2	hST3 A2
ST3A1	hST3 A2
ST3H6	zST3
ST2	hST6Gal2

Posthoc Non-parametric test:

Multiple Comparisons

Tamhane

Dependent Variable	(I) Sample	(J) Sample	Mean Difference (I-J)	Std. Error	Sig.	95% Confidence Interval	
						Lower Bound	Upper Bound
SNA-I	A5	ASF	.17057	.026836	.881	-.81374	1.15488
		B1	-.61080	.050248	.072	-1.29693	.07533
		Ctrl	-1.86787	.092622	.109	-4.50383	.76810
		Dre	-.30683	.042418	.219	-.77809	.16443
		FET	-2.09023	.109416	.152	-5.60628	1.42581
		G1	.11367	.035482	.971	-.25962	.48695
		Gac	-.03847	.085478	1.000	-2.30482	2.22789
		Gga	-1.78183*	.069087	.030	-3.23693	-.32674
		Rno	-1.42407	.077736	.097	-3.29835	.45021
		ST2	.18497	.040800	.694	-.25365	.62359
		ST3A1	-.02837	.032870	1.000	-.40905	.35232
		ST3A2	.06663	.046160	1.000	-.49666	.62993
		ST3H6	-.05930	.050348	1.000	-.74870	.63010
		Tru	-.41667	.067462	.705	-1.79620	.96287

	ASF	A5	-.17057	.026836	.881	-1.15488	.81374
		B1	-.78137	.043064	.245	-2.57315	1.01041
		Ctrl	-2.03843	.088929	.176	-5.98199	1.90512
		Dre	-.47740	.033600	.351	-1.80424	.84944
		FET	-2.26080	.106309	.203	-7.00313	2.48153
		G1	-.05690	.024261	1.000	-.90809	.79429
		Gac	-.20903	.081463	1.000	-3.80784	3.38977
		Gga	-1.95240	.064052	.100	-4.74137	.83657
		Rno	-1.59463	.073297	.191	-4.81486	1.62559
		ST2	.01440	.031532	1.000	-1.20879	1.23759
		ST3A1	-.19893	.020251	.508	-.84038	.44251
		ST3A2	-.10393	.038215	1.000	-1.65923	1.45136
		ST3H6	-.22987	.043180	.966	-2.02730	1.56757
		Tru	-.58723	.062295	.674	-3.29386	2.11939
	B1	A5	.61080	.050248	.072	-.07533	1.29693
		ASF	.78137	.043064	.245	-1.01041	2.57315
		Ctrl	-1.25707	.098555	.125	-3.01349	.49936
		Dre	.30397	.054163	.463	-.30557	.91350
		FET	-1.47943	.114482	.179	-3.95358	.99472
		G1	.72447	.048921	.055	-.02120	1.47013
		Gac	.57233	.091874	.575	-.91280	2.05747
		Gga	-1.17103*	.076859	.026	-2.14065	-.20141
		Rno	-.81327	.084718	.169	-2.03425	.40771
		ST2	.79577*	.052905	.021	.17289	1.41864
		ST3A1	.58243	.047061	.151	-.30258	1.46745
		ST3A2	.67743*	.057140	.032	.07336	1.28151
		ST3H6	.55150	.060574	.081	-.07659	1.17959
		Tru	.19413	.075402	.999	-.73405	1.12231
	Ctrl	A5	1.86787	.092622	.109	-.76810	4.50383
		ASF	2.03843	.088929	.176	-1.90512	5.98199
		B1	1.25707	.098555	.125	-.49936	3.01349
		Dre	1.56103	.094803	.116	-.64473	3.76680
		FET	-.22237	.138420	1.000	-1.71892	1.27418
		G1	1.98153	.091909	.108	-.83725	4.80032
		Gac	1.82940*	.120394	.012	.56820	3.09060
		Gga	.08603	.109368	1.000	-1.21357	1.38563
		Rno	.44380	.115026	.873	-.80882	1.69642
		ST2	2.05283	.094090	.068	-.27543	4.38110
		ST3A1	1.83950	.090932	.150	-1.27465	4.95365
		ST3A2	1.93450	.096535	.052	-.02934	3.89834
		ST3H6	1.80857*	.098606	.046	.05657	3.56056
		Tru	1.45120*	.108348	.036	.13437	2.76803

	Dre	A5	.30683	.042418	.219	-.16443	.77809
		ASF	.47740	.033600	.351	-.84944	1.80424
		B1	-.30397	.054163	.463	-.91350	.30557
		Ctrl	-1.56103	.094803	.116	-3.76680	.64473
		FET	-1.78340	.111268	.167	-4.82239	1.25559
		G1	.42050	.040838	.083	-.06726	.90826
		Gac	.26837	.087837	.999	-1.60179	2.13852
		Gga	-1.47500*	.071985	.026	-2.65278	-.29722
		Rno	-1.11723	.080322	.115	-2.64400	.40954
		ST2	.49180*	.045535	.044	.01700	.96660
		ST3A1	.27847	.038591	.357	-.27205	.82898
		ST3A2	.37347	.050394	.180	-.16103	.90797
		ST3H6	.24753	.054256	.715	-.36410	.85917
		Tru	-.10983	.070426	1.000	-1.22709	1.00742
	FET	A5	2.09023	.109416	.152	-1.42581	5.60628
		ASF	2.26080	.106309	.203	-2.48153	7.00313
		B1	1.47943	.114482	.179	-.99472	3.95358
		Ctrl	.22237	.138420	1.000	-1.27418	1.71892
		Dre	1.78340	.111268	.167	-1.25559	4.82239
		G1	2.20390	.108813	.148	-1.50127	5.90907
		Gac	2.05177*	.133746	.017	.53261	3.57093
		Gga	.30840	.123913	1.000	-1.42682	2.04362
		Rno	.66617	.128935	.620	-.92140	2.25373
		ST2	2.27520	.110662	.106	-.90481	5.45521
		ST3A1	2.06187	.107990	.190	-1.93489	6.05863
		ST3A2	2.15687	.112748	.089	-.58872	4.90245
		ST3H6	2.03093	.114526	.082	-.43717	4.49904
		Tru	1.67357	.123014	.060	-.09990	3.44704
	G1	A5	-.11367	.035482	.971	-.48695	.25962
		ASF	.05690	.024261	1.000	-.79429	.90809
		B1	-.72447	.048921	.055	-1.47013	.02120
		Ctrl	-1.98153	.091909	.108	-4.80032	.83725
		Dre	-.42050	.040838	.083	-.90826	.06726
		FET	-2.20390	.108813	.148	-5.90907	1.50127
		Gac	-.15213	.084705	1.000	-2.59375	2.28948
		Gga	-1.89550*	.068128	.033	-3.49070	-.30030
		Rno	-1.53773	.076885	.096	-3.57424	.49877
		ST2	.07130	.039154	1.000	-.37465	.51725
		ST3A1	-.14203	.030804	.680	-.47687	.19281
		ST3A2	-.04703	.044712	1.000	-.64770	.55363
		ST3H6	-.17297	.049024	.979	-.92242	.57649
		Tru	-.53033	.066479	.522	-2.04479	.98413

	Gac	A5	.03847	.085478	1.000	-2.22789	2.30482
		ASF	.20903	.081463	1.000	-3.38977	3.80784
		B1	-.57233	.091874	.575	-2.05747	.91280
		Ctrl	-1.82940*	.120394	.012	-3.09060	-.56820
		Dre	-.26837	.087837	.999	-2.13852	1.60179
		FET	-2.05177*	.133746	.017	-3.57093	-.53261
		G1	.15213	.084705	1.000	-2.28948	2.59375
		Gga	-1.74337*	.103388	.011	-2.89907	-.58767
		Rno	-1.38560*	.109356	.025	-2.53646	-.23474
		ST2	.22343	.087067	1.000	-1.75709	2.20396
		ST3A1	.01010	.083645	1.000	-2.72262	2.74282
		ST3A2	.10510	.089703	1.000	-1.55351	1.76371
		ST3H6	-.02083	.091929	1.000	-1.50236	1.46069
		Tru	-.37820	.102309	.918	-1.54229	.78589
	Gga	A5	1.78183*	.069087	.030	.32674	3.23693
		ASF	1.95240	.064052	.100	-.83657	4.74137
		B1	1.17103*	.076859	.026	.20141	2.14065
		Ctrl	-.08603	.109368	1.000	-1.38563	1.21357
		Dre	1.47500*	.071985	.026	.29722	2.65278
		FET	-.30840	.123913	1.000	-2.04362	1.42682
		G1	1.89550*	.068128	.033	.30030	3.49070
		Gac	1.74337*	.103388	.011	.58767	2.89907
		Rno	.35777	.097084	.901	-.67350	1.38903
		ST2	1.96680*	.071043	.014	.71746	3.21614
		ST3A1	1.75347	.066805	.057	-.09759	3.60453
		ST3A2	1.84847*	.074251	.008	.79415	2.90279
		ST3H6	1.72253*	.076925	.007	.75447	2.69060
		Tru	1.36517*	.089071	.011	.44065	2.28969
	Rno	A5	1.42407	.077736	.097	-.45021	3.29835
		ASF	1.59463	.073297	.191	-1.62559	4.81486
		B1	.81327	.084718	.169	-.40771	2.03425
		Ctrl	-.44380	.115026	.873	-1.69642	.80882
		Dre	1.11723	.080322	.115	-.40954	2.64400
		FET	-.66617	.128935	.620	-2.25373	.92140
		G1	1.53773	.076885	.096	-.49877	3.57424
		Gac	1.38560*	.109356	.025	.23474	2.53646
		Gga	-.35777	.097084	.901	-1.38903	.67350
		ST2	1.60903	.079479	.051	-.01167	3.22973
		ST3A1	1.39570	.075715	.150	-.92073	3.71213
		ST3A2	1.49070*	.082359	.038	.13676	2.84464
		ST3H6	1.36477*	.084777	.035	.14644	2.58309
		Tru	1.00740	.095934	.054	-.02277	2.03757

	ST2	A5	-.18497	.040800	.694	-.62359	.25365
		ASF	-.01440	.031532	1.000	-1.23759	1.20879
		B1	-.79577*	.052905	.021	-1.41864	-.17289
		Ctrl	-2.05283	.094090	.068	-4.38110	.27543
		Dre	-.49180*	.045535	.044	-.96660	-.01700
		FET	-2.27520	.110662	.106	-5.45521	.90481
		G1	-.07130	.039154	1.000	-.51725	.37465
		Gac	-.22343	.087067	1.000	-2.20396	1.75709
		Gga	-1.96680*	.071043	.014	-3.21614	-.71746
		Rno	-1.60903	.079479	.051	-3.22973	.01167
		ST3A1	-.21333	.036805	.538	-.70491	.27825
		ST3A2	-.11833	.049039	1.000	-.65295	.41628
		ST3H6	-.24427	.053000	.729	-.86957	.38103
		Tru	-.60163	.069464	.306	-1.78583	.58256
	ST3A1	A5	.02837	.032870	1.000	-.35232	.40905
		ASF	.19893	.020251	.508	-.44251	.84038
		B1	-.58243	.047061	.151	-1.46745	.30258
		Ctrl	-1.83950	.090932	.150	-4.95365	1.27465
		Dre	-.27847	.038591	.357	-.82898	.27205
		FET	-2.06187	.107990	.190	-6.05863	1.93489
		G1	.14203	.030804	.680	-.19281	.47687
		Gac	-.01010	.083645	1.000	-2.74282	2.72262
		Gga	-1.75347	.066805	.057	-3.60453	.09759
		Rno	-1.39570	.075715	.150	-3.71213	.92073
		ST2	.21333	.036805	.538	-.27825	.70491
		ST3A2	.09500	.042669	1.000	-.60659	.79659
		ST3H6	-.03093	.047168	1.000	-.92063	.85876
		Tru	-.38830	.065123	.840	-2.15236	1.37576
	ST3A2	A5	-.06663	.046160	1.000	-.62993	.49666
		ASF	.10393	.038215	1.000	-1.45136	1.65923
		B1	-.67743*	.057140	.032	-1.28151	-.07336
		Ctrl	-1.93450	.096535	.052	-3.89834	.02934
		Dre	-.37347	.050394	.180	-.90797	.16103
		FET	-2.15687	.112748	.089	-4.90245	.58872
		G1	.04703	.044712	1.000	-.55363	.64770
		Gac	-.10510	.089703	1.000	-1.76371	1.55351
		Gga	-1.84847*	.074251	.008	-2.90279	-.79415
		Rno	-1.49070*	.082359	.038	-2.84464	-.13676
		ST2	.11833	.049039	1.000	-.41628	.65295
		ST3A1	-.09500	.042669	1.000	-.79659	.60659
		ST3H6	-.12593	.057228	1.000	-.73147	.47961
		Tru	-.48330	.072741	.415	-1.48693	.52033

	ST3H6	A5	.05930	.050348	1.000	-.63010	.74870
		ASF	.22987	.043180	.966	-1.56757	2.02730
		B1	-.55150	.060574	.081	-1.17959	.07659
		Ctrl	-1.80857*	.098606	.046	-3.56056	-.05657
		Dre	-.24753	.054256	.715	-.85917	.36410
		FET	-2.03093	.114526	.082	-4.49904	.43717
		G1	.17297	.049024	.979	-.57649	.92242
		Gac	.02083	.091929	1.000	-1.46069	1.50236
		Gga	-1.72253*	.076925	.007	-2.69060	-.75447
		Rno	-1.36477*	.084777	.035	-2.58309	-.14644
		ST2	.24427	.053000	.729	-.38103	.86957
		ST3A1	.03093	.047168	1.000	-.85876	.92063
		ST3A2	.12593	.057228	1.000	-.47961	.73147
		Tru	-.35737	.075468	.720	-1.28420	.56946
	Tru	A5	.41667	.067462	.705	-.96287	1.79620
		ASF	.58723	.062295	.674	-2.11939	3.29386
		B1	-.19413	.075402	.999	-1.12231	.73405
		Ctrl	-1.45120*	.108348	.036	-2.76803	-.13437
		Dre	.10983	.070426	1.000	-1.00742	1.22709
		FET	-1.67357	.123014	.060	-3.44704	.09990
		G1	.53033	.066479	.522	-.98413	2.04479
		Gac	.37820	.102309	.918	-.78589	1.54229
		Gga	-1.36517*	.089071	.011	-2.28969	-.44065
		Rno	-1.00740	.095934	.054	-2.03757	.02277
		ST2	.60163	.069464	.306	-.58256	1.78583
		ST3A1	.38830	.065123	.840	-1.37576	2.15236
		ST3A2	.48330	.072741	.415	-.52033	1.48693
		ST3H6	.35737	.075468	.720	-.56946	1.28420
RCA-I	A5	ASF	-.61620	.085466	.201	-1.52969	.29729
		B1	.05757	.074180	1.000	-.72829	.84343
		Ctrl	.08533	.062245	1.000	-.98042	1.15108
		Dre	-.11973	.100160	1.000	-1.38494	1.14547
		FET	-.25147	.067045	.933	-1.09650	.59357
		G1	-.02910	.121842	1.000	-2.05058	1.99238
		Gac	-.13757	.078188	1.000	-.94851	.67338
		Gga	.10380	.080815	1.000	-.73699	.94459
		Rno	-.05687	.074035	1.000	-.84239	.72865
		ST2	-.56807	.073609	.159	-1.35286	.21672
		ST3A1	-.02160	.107392	1.000	-1.51258	1.46938
		ST3A2	-.16010	.060565	1.000	-1.38921	1.06901
		ST3H6	-.11453	.162259	1.000	-3.97794	3.74887
		Tru	-.18683	.062283	.998	-1.24965	.87598

	ASF	A5	.61620	.085466	.201	-.29729	1.52969
		B1	.67377	.081273	.152	-.25740	1.60493
		Ctrl	.70153	.070549	.292	-.72560	2.12867
		Dre	.49647	.105521	.676	-.68919	1.68212
		FET	.36473	.074818	.778	-.73143	1.46089
		G1	.58710	.126286	.806	-1.19074	2.36494
		Gac	.47863	.084947	.427	-.43474	1.39201
		Gga	.72000	.087371	.122	-.19839	1.63839
		Rno	.55933	.081141	.273	-.37320	1.49187
		ST2	.04813	.080753	1.000	-.88875	.98502
		ST3A1	.59460	.112409	.579	-.76092	1.95012
		ST3A2	.45610	.069071	.711	-1.18013	2.09233
		ST3H6	.50167	.165622	.999	-2.87860	3.88194
		Tru	.42937	.070582	.713	-.99383	1.85256
	B1	A5	-.05757	.074180	1.000	-.84343	.72829
		ASF	-.67377	.081273	.152	-1.60493	.25740
		Ctrl	.02777	.056349	1.000	-.81083	.86636
		Dre	-.17730	.096607	1.000	-1.56038	1.20578
		FET	-.30903	.061611	.606	-1.01115	.39309
		G1	-.08667	.118939	1.000	-2.35932	2.18599
		Gac	-.19513	.073581	.998	-.97110	.58083
		Gga	.04623	.076367	1.000	-.77913	.87159
		Rno	-.11443	.069152	1.000	-.83148	.60261
		ST2	-.62563	.068696	.081	-1.33823	.08697
		ST3A1	-.07917	.104086	1.000	-1.73519	1.57686
		ST3A2	-.21767	.054488	.959	-1.17939	.74406
		ST3H6	-.17210	.160090	1.000	-4.44705	4.10285
		Tru	-.24440	.056391	.883	-1.08090	.59210
	Ctrl	A5	-.08533	.062245	1.000	-1.15108	.98042
		ASF	-.70153	.070549	.292	-2.12867	.72560
		B1	-.02777	.056349	1.000	-.86636	.81083
		Dre	-.20507	.087776	1.000	-2.47362	2.06349
		FET	-.33680	.046558	.240	-.87573	.20213
		G1	-.11443	.111885	1.000	-3.63800	3.40914
		Gac	-.22290	.061530	.981	-1.25961	.81381
		Gga	.01847	.064836	1.000	-1.15554	1.19247
		Rno	-.14220	.056158	1.000	-.97394	.68954
		ST2	-.65340	.055596	.097	-1.46518	.15838
		ST3A1	-.10693	.095946	1.000	-2.79632	2.58245
		ST3A2	-.24543	.036616	.255	-.63726	.14640
		ST3H6	-.19987	.154921	1.000	-5.96045	5.56071
		Tru	-.27217	.039392	.215	-.68062	.13629

	Dre	A5	.11973	.100160	1.000	-1.14547	1.38494
		ASF	-.49647	.105521	.676	-1.68212	.68919
		B1	.17730	.096607	1.000	-1.20578	1.56038
		Ctrl	.20507	.087776	1.000	-2.06349	2.47362
		FET	-.13173	.091243	1.000	-1.89097	1.62751
		G1	.09063	.136660	1.000	-1.45829	1.63955
		Gac	-.01783	.099717	1.000	-1.29416	1.25850
		Gga	.22353	.101791	1.000	-1.00761	1.45467
		Rno	.06287	.096495	1.000	-1.32514	1.45087
		ST2	-.44833	.096169	.818	-1.85129	.95462
		ST3A1	.09813	.123949	1.000	-1.20349	1.39975
		ST3A2	-.04037	.086593	1.000	-2.57442	2.49369
		ST3H6	.00520	.173661	1.000	-2.68817	2.69857
		Tru	-.06710	.087803	1.000	-2.33034	2.19614
	FET	A5	.25147	.067045	.933	-.59357	1.09650
		ASF	-.36473	.074818	.778	-1.46089	.73143
		B1	.30903	.061611	.606	-.39309	1.01115
		Ctrl	.33680	.046558	.240	-.20213	.87573
		Dre	.13173	.091243	1.000	-1.62751	1.89097
		G1	.22237	.114625	1.000	-2.65996	3.10469
		Gac	.11390	.066382	1.000	-.71212	.93992
		Gga	.35527	.069457	.674	-.56231	1.27285
		Rno	.19460	.061436	.981	-.50344	.89264
		ST2	-.31660	.060923	.555	-1.00287	.36967
		ST3A1	.22987	.099128	1.000	-1.89165	2.35139
		ST3A2	.09137	.044287	1.000	-.49341	.67615
		ST3H6	.13693	.156912	1.000	-4.94554	5.21941
		Tru	.06463	.046609	1.000	-.47372	.60298
	G1	A5	.02910	.121842	1.000	-1.99238	2.05058
		ASF	-.58710	.126286	.806	-2.36494	1.19074
		B1	.08667	.118939	1.000	-2.18599	2.35932
		Ctrl	.11443	.111885	1.000	-3.40914	3.63800
		Dre	-.09063	.136660	1.000	-1.63955	1.45829
		FET	-.22237	.114625	1.000	-3.10469	2.65996
		Gac	-.10847	.121479	1.000	-2.15653	1.93960
		Gga	.13290	.123186	1.000	-1.80023	2.06603
		Rno	-.02777	.118848	1.000	-2.30986	2.25433
		ST2	-.53897	.118584	.921	-2.84934	1.77141
		ST3A1	.00750	.142045	1.000	-1.51921	1.53421
		ST3A2	-.13100	.110959	1.000	-3.94205	3.68005
		ST3H6	-.08543	.187008	1.000	-2.32765	2.15678
		Tru	-.15773	.111906	1.000	-3.67527	3.35980

	Gac	A5	.13757	.078188	1.000	-.67338	.94851
		ASF	-.47863	.084947	.427	-1.39201	.43474
		B1	.19513	.073581	.998	-.58083	.97110
		Ctrl	.22290	.061530	.981	-.81381	1.25961
		Dre	.01783	.099717	1.000	-1.25850	1.29416
		FET	-.11390	.066382	1.000	-.93992	.71212
		G1	.10847	.121479	1.000	-1.93960	2.15653
		Gga	.24137	.080266	.986	-.59556	1.07829
		Rno	.08070	.073434	1.000	-.69478	.85618
		ST2	-.43050	.073006	.368	-1.20481	.34381
		ST3A1	.11597	.106979	1.000	-1.39151	1.62344
		ST3A2	-.02253	.059830	1.000	-1.21806	1.17299
		ST3H6	.02303	.161986	1.000	-3.88719	3.93326
		Tru	-.04927	.061568	1.000	-1.08314	.98461
	Gga	A5	-.10380	.080815	1.000	-.94459	.73699
		ASF	-.72000	.087371	.122	-1.63839	.19839
		B1	-.04623	.076367	1.000	-.87159	.77913
		Ctrl	-.01847	.064836	1.000	-1.19247	1.15554
		Dre	-.22353	.101791	1.000	-1.45467	1.00761
		FET	-.35527	.069457	.674	-1.27285	.56231
		G1	-.13290	.123186	1.000	-2.06603	1.80023
		Gac	-.24137	.080266	.986	-1.07829	.59556
		Rno	-.16067	.076226	1.000	-.98622	.66489
		ST2	-.67187	.075813	.105	-1.49828	.15455
		ST3A1	-.12540	.108914	1.000	-1.56361	1.31281
		ST3A2	-.26390	.063225	.967	-1.61696	1.08916
		ST3H6	-.21833	.163271	1.000	-3.91910	3.48243
		Tru	-.29063	.064872	.912	-1.46137	.88010
	Rno	A5	.05687	.074035	1.000	-.72865	.84239
		ASF	-.55933	.081141	.273	-1.49187	.37320
		B1	.11443	.069152	1.000	-.60261	.83148
		Ctrl	.14220	.056158	1.000	-.68954	.97394
		Dre	-.06287	.096495	1.000	-1.45087	1.32514
		FET	-.19460	.061436	.981	-.89264	.50344
		G1	.02777	.118848	1.000	-2.25433	2.30986
		Gac	-.08070	.073434	1.000	-.85618	.69478
		Gga	.16067	.076226	1.000	-.66489	.98622
		ST2	-.51120	.068539	.166	-1.22204	.19964
		ST3A1	.03527	.103983	1.000	-1.62732	1.69786
		ST3A2	-.10323	.054290	1.000	-1.05670	.85023
		ST3H6	-.05767	.160023	1.000	-4.34693	4.23159
		Tru	-.12997	.056200	1.000	-.95964	.69970

	ST2	A5	.56807	.073609	.159	-.21672	1.35286
		ASF	-.04813	.080753	1.000	-.98502	.88875
		B1	.62563	.068696	.081	-.08697	1.33823
		Ctrl	.65340	.055596	.097	-.15838	1.46518
		Dre	.44833	.096169	.818	-.95462	1.85129
		FET	.31660	.060923	.555	-.36967	1.00287
		G1	.53897	.118584	.921	-1.77141	2.84934
		Gac	.43050	.073006	.368	-.34381	1.20481
		Gga	.67187	.075813	.105	-.15455	1.49828
		Rno	.51120	.068539	.166	-.19964	1.22204
		ST3A1	.54647	.103680	.749	-1.13592	2.22885
		ST3A2	.40797	.053709	.420	-.52139	1.33732
		ST3H6	.45353	.159827	1.000	-3.87815	4.78522
		Tru	.38123	.055638	.417	-.42856	1.19103
	ST3A1	A5	.02160	.107392	1.000	-1.46938	1.51258
		ASF	-.59460	.112409	.579	-1.95012	.76092
		B1	.07917	.104086	1.000	-1.57686	1.73519
		Ctrl	.10693	.095946	1.000	-2.58245	2.79632
		Dre	-.09813	.123949	1.000	-1.39975	1.20349
		FET	-.22987	.099128	1.000	-2.35139	1.89165
		G1	-.00750	.142045	1.000	-1.53421	1.51921
		Gac	-.11597	.106979	1.000	-1.62344	1.39151
		Gga	.12540	.108914	1.000	-1.31281	1.56361
		Rno	-.03527	.103983	1.000	-1.69786	1.62732
		ST2	-.54647	.103680	.749	-2.22885	1.13592
		ST3A2	-.13850	.094865	1.000	-3.10625	2.82925
		ST3H6	-.09293	.177931	1.000	-2.57959	2.39372
		Tru	-.16523	.095971	1.000	-2.84894	2.51847
	ST3A2	A5	.16010	.060565	1.000	-1.06901	1.38921
		ASF	-.45610	.069071	.711	-2.09233	1.18013
		B1	.21767	.054488	.959	-.74406	1.17939
		Ctrl	.24543	.036616	.255	-.14640	.63726
		Dre	.04037	.086593	1.000	-2.49369	2.57442
		FET	-.09137	.044287	1.000	-.67615	.49341
		G1	.13100	.110959	1.000	-3.68005	3.94205
		Gac	.02253	.059830	1.000	-1.17299	1.21806
		Gga	.26390	.063225	.967	-1.08916	1.61696
		Rno	.10323	.054290	1.000	-.85023	1.05670
		ST2	-.40797	.053709	.420	-1.33732	.52139
		ST3A1	.13850	.094865	1.000	-2.82925	3.10625
		ST3H6	.04557	.154254	1.000	-5.98140	6.07253
		Tru	-.02673	.036680	1.000	-.41973	.36627

ST3H6	A5	.11453	.162259	1.000	-3.74887	3.97794
	ASF	-.50167	.165622	.999	-3.88194	2.87860
	B1	.17210	.160090	1.000	-4.10285	4.44705
	Ctrl	.19987	.154921	1.000	-5.56071	5.96045
	Dre	-.00520	.173661	1.000	-2.69857	2.68817
	FET	-.13693	.156912	1.000	-5.21941	4.94554
	G1	.08543	.187008	1.000	-2.15678	2.32765
	Gac	-.02303	.161986	1.000	-3.93326	3.88719
	Gga	.21833	.163271	1.000	-3.48243	3.91910
	Rno	.05767	.160023	1.000	-4.23159	4.34693
	ST2	-.45353	.159827	1.000	-4.78522	3.87815
	ST3A1	.09293	.177931	1.000	-2.39372	2.57959
	ST3A2	-.04557	.154254	1.000	-6.07253	5.98140
	Tru	-.07230	.154937	1.000	-5.82708	5.68248
Tru	A5	.18683	.062283	.998	-.87598	1.24965
	ASF	-.42937	.070582	.713	-1.85256	.99383
	B1	.24440	.056391	.883	-.59210	1.08090
	Ctrl	.27217	.039392	.215	-.13629	.68062
	Dre	.06710	.087803	1.000	-2.19614	2.33034
	FET	-.06463	.046609	1.000	-.60298	.47372
	G1	.15773	.111906	1.000	-3.35980	3.67527
	Gac	.04927	.061568	1.000	-.98461	1.08314
	Gga	.29063	.064872	.912	-.88010	1.46137
	Rno	.12997	.056200	1.000	-.69970	.95964
	ST2	-.38123	.055638	.417	-1.19103	.42856
	ST3A1	.16523	.095971	1.000	-2.51847	2.84894
	ST3A2	.02673	.036680	1.000	-.36627	.41973
	ST3H6	.07230	.154937	1.000	-5.68248	5.82708

Based on observed means.

The error term is Mean Square(Error) = .015.

*. The mean difference is significant at the .05 level.
